# Introducing Chirality into Graphene Oxide Layers through a Facile Amino Acid-Based Covalent Functionalization

**DOI:** 10.1021/acsomega.6c07448

**Published:** 2026-10-26

**Authors:** Francesco Amato, Samuele De Santis, Matteo Daino, Leonardo Giaccari, Alessandro Motta, Mauro Giustini, Francesca Anna Scaramuzzo, Anita Scipioni, Andrea Giacomo Marrani

**Affiliations:** † Dipartimento di Chimica, Sapienza Università di Roma, P.le A. Moro 5, I-00185 Rome, Italy; ‡ Department for Sustainability, ENEA, Casaccia Research Centre, I-00123 Rome, Italy; § INSTM Consortium, UdR Roma, P.le A. Moro 5, I-00185 Rome, Italy; ∥ Dipartimento di Scienze di Base e Applicate per l’Ingegneria (S.B.A.I.), Sapienza Università di Roma, Via del Castro Laurenziano 7, I-00161 Rome, Italy

## Abstract

Graphene-based materials, including graphene oxide (GO), intrinsically lack stereocenters, limiting their usefulness in applications where chirality plays a crucial role. Covalent attachment of chiral molecules offers a promising strategy to introduce stereogenic elements into layers of GO. In this work, we report a one-pot method for the covalent functionalization of GO with lysine, exploiting the reactivity of the aminic groups of the amino acid toward epoxy groups located on the basal plane of the layers. Chirally functionalized layers of GO were prepared using d- and l-lysine and extensively purified to remove unreacted amino acid residuals. A comprehensive spectroscopic characterization confirms the successful functionalization, resulting in ∼3% N content. The chirality of the molecules bound to the resulting GO layers (relying on a rigorous purification by dialysis) was demonstrated through their interaction with a water-soluble porphyrin, which yielded chiral J-aggregates. The corresponding circular dichroism spectra confirmed that the porphyrin assemblies adopt distinct chiral configurations when templated by d- or l-GO. These findings highlight a straightforward route to impart chiral features to GO, expanding its applicability to several relevant fields, such as asymmetric catalysis, nanofiltration, and chiral sensing.

## Graphical abstract

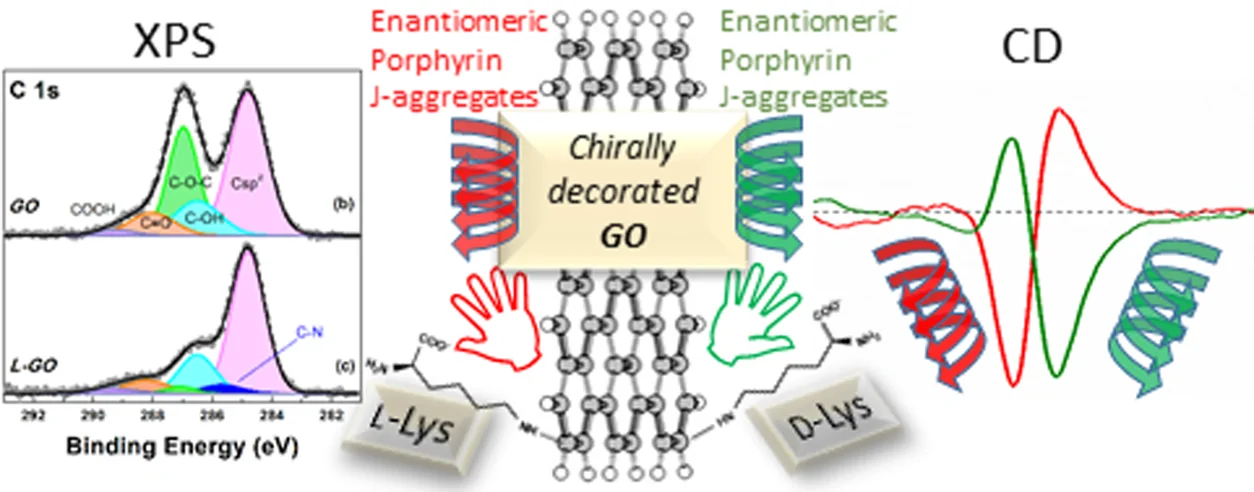

## Introduction

Chirality is a fundamental physical property exhibited by many molecules that plays a crucial role throughout nature. Biological macromolecules such as proteins, sugars, and DNA contain numerous stereocenters, and their functions are intimately linked to their chiral structures. This same property is equally important in pharmaceutical chemistry because many therapeutic compounds are chiral and their biological activity and efficacy can vary dramatically between different enantiomers.

In recent years, interest in the synthesis of chiral nanostructures, both inorganic and organic, has grown considerably. Chirality at the nanoscale opens pathways to a wide range of advanced applications, including asymmetric catalysis, biosensing, chiroptical or optoelectronic technologies, as well as the modulation and regulation of biological processes.^1–5^ Among the various strategies for producing such materials, bottom-up approaches are particularly powerful. These methods rely on asymmetric templates and chiral reagents to direct the formation of intrinsically chiral inorganic and organic nanoparticles, respectively.^6^ In addition, chirality can be introduced postsynthetically. Specifically, stereogenic molecules can be grafted onto nanoparticle surfaces through covalent coupling, enabling the efficient transfer of molecular chirality at the nanostructure level.^7,8^

Graphene oxide (GO) is a two-dimensional carbon-based material composed primarily of sp^2^-hybridized carbon atom layers decorated with abundant oxygen-containing functional groups. Hydroxyl and epoxy groups are predominantly located on the basal planes, while less-abundant carboxyl functionalities are confined to the sheet edges.^9^ Since GO is typically produced by chemical oxidation of graphite, it inherently lacks asymmetric carbon centers and is therefore achiral.

Among the different strategies developed to introduce chirality into GO layers, covalent surface functionalization with chiral molecules has emerged as the most widely explored approach. In particular, chiral modifiers such as cyclodextrins and amino acids have been successfully anchored onto GO surfaces to endow the material with stereochemical features.^10–13^ Amino acids have proven especially versatile in this context as they can react with GO either through amide-bond formation in the presence of coupling agents or via one-pot procedures that exploit the nucleophilic ring opening of epoxy groups by the amino functionalities of amino acids, in addition to a covalent grafting via the thiol–ene click mechanism in the case of cysteine.^14–18^

Various amino acids, including l-lysine, l-glutamic acid, l-methionine, and l-arginine, have been covalently grafted onto GO surfaces through epoxide ring-opening reactions that selectively involve their α-amino groups. These functionalization reactions were carried out in aqueous media at pH = 9, and the resulting amino-functionalized GO materials were purified using an innovative microfiltration-based method, as demonstrated in several recent studies.^15,16,18^ In contrast, Z. Mo et al. reported a different strategy in which l-lysine was grafted onto GO in the form of a Cu­(l-Lys)_2_ coordination complex. In this system, the Cu^2+^ ions are coordinated by the α-carboxylate and α-amino groups of l-lysine, thereby leaving the ε-amine side-chain group available to react with the epoxide groups of GO.^19^ Finally, in another study, d- and l-cysteine were covalently grafted onto GO via hydrothermal treatment at 120 °C for 16 h, yielding materials reported as enantiomeric layers, as shown by circular dichroism (CD) spectra. The authors attributed the observed CD signals to the intrinsic chirality of the functionalized GO, given that the CD spectra of d- and l-GO closely resembled those of the corresponding d- and l-cysteine precursors.^20^ However, it is to be noted that the possible presence of residual, unbound amino acid molecules could contribute to—or even dominate—the CD response, complicating the assignment of chirality to the GO itself. A comparison among the different grafting methods used in terms of functionalization degree is reported in Table S1.

In this work, we report the one-pot covalent grafting of d- and l-lysine onto GO to obtain chirally functionalized GO layers. The functionalization reactions were carried out at pH > 10, *i*.*e*., around the p*K*_a_ value of the ε-amino group of the lysine side chain. The resulting crude products were extensively purified via dialysis to ensure the complete removal of unreacted lysine residuals and salts. The purified lysine-functionalized GO layers were then characterized spectroscopically, and the functionalization reaction mechanism was elucidated through DFT calculations. Subsequently, we report the investigation on the chirality of d- and l-GO in the presence of the water-soluble 5,10,15,20-(tetra-4-sulfonatophenyl)­porphyrin (TPPS), a porphyrin derivative known to form chiral J-aggregates when exposed to chiral inducers.^21,22^ The macromolecular interaction between porphyrins and GO, and its derivatives, arises primarily from two major contributions that can be rationalized based on their molecular structures: electrostatic interactions and weak dispersion forces, particularly π–π stacking. These interactions occur because both species contain charged functional groups as well as extended aromatic and conjugated domains.^23,24^ After optimizing the aggregating conditions, J-aggregates formed in the presence of d- and l-GO were isolated and morphologically characterized, while their chiroptical properties were studied by CD spectroscopy. Our study aims to demonstrate the chiral characteristics of GO covalently grafted with lysine, paving the way for the application of these nanomaterials in asymmetric catalysis, nanofiltration, and chiral sensing.

## Results and Discussion

### Amino Functionalization of GO with l-Lysine (l-GO)

The chemical derivatization of GO with l-lysine, which resulted in the l-GO sample, was carried out under mild experimental conditions, as illustrated in Scheme 1. AFM analysis on pristine GO (Figure S1) showed the presence of nearly one-atom-thick layers. This sample was dispersed in water at pH = 10.5, where a significant fraction of the α- and ε-amino groups of l-lysine is present in the neutral (unprotonated) form, enabling their reaction with the epoxide groups of GO.^25^

**Scheme 1:**
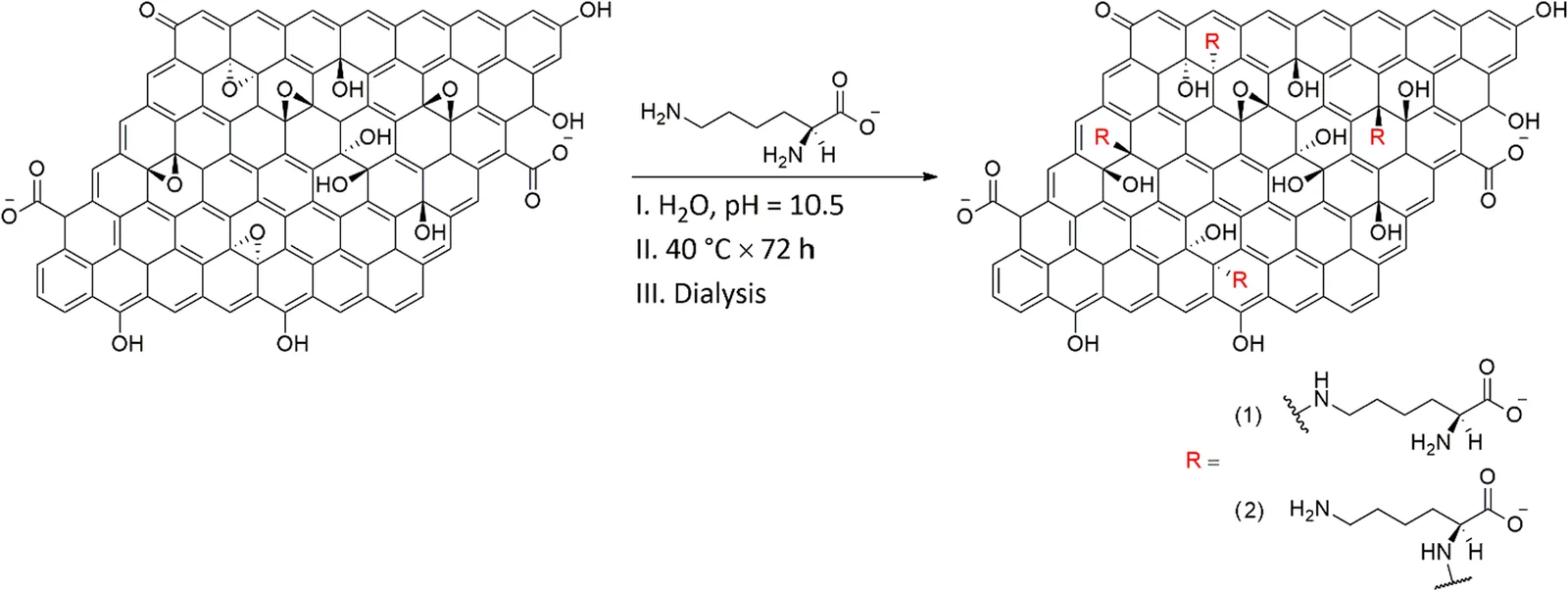
Functionalization of GO in the Presence of l-Lysine^a^

a Both GO-bound isomers deriving from the reaction of (1) ε- and (2) α-amino groups are reported.

Moreover, this pH value was specifically selected because, at substantially higher pH, GO is known to undergo severe deoxygenation, although such processes may already begin at pH ∼ 10.5.^26^ In the UV–vis spectrum of l-GO (Figure 1a, red line), the typical π–π* and n–π* absorption bands at ∼230 and ∼300 nm are slightly red-shifted and attenuated compared to GO (Figure 1a, black line). In addition, l-GO displays an increased absorption in the visible region, similar to previous observations of deoxygenated GO, suggesting that partial disproportion occurred during functionalization with lysine.^26^ Covalent grafting of l-lysine onto the GO layers was initially evaluated using the colorimetric Kaiser test (KT, Figure 1b), performed at *T* = 120 °C, which revealed the presence of 120 ± 20 μmol g^–1^ of primary aliphatic amines in the structure of l-GO.^27,28^

**Figure 1:**
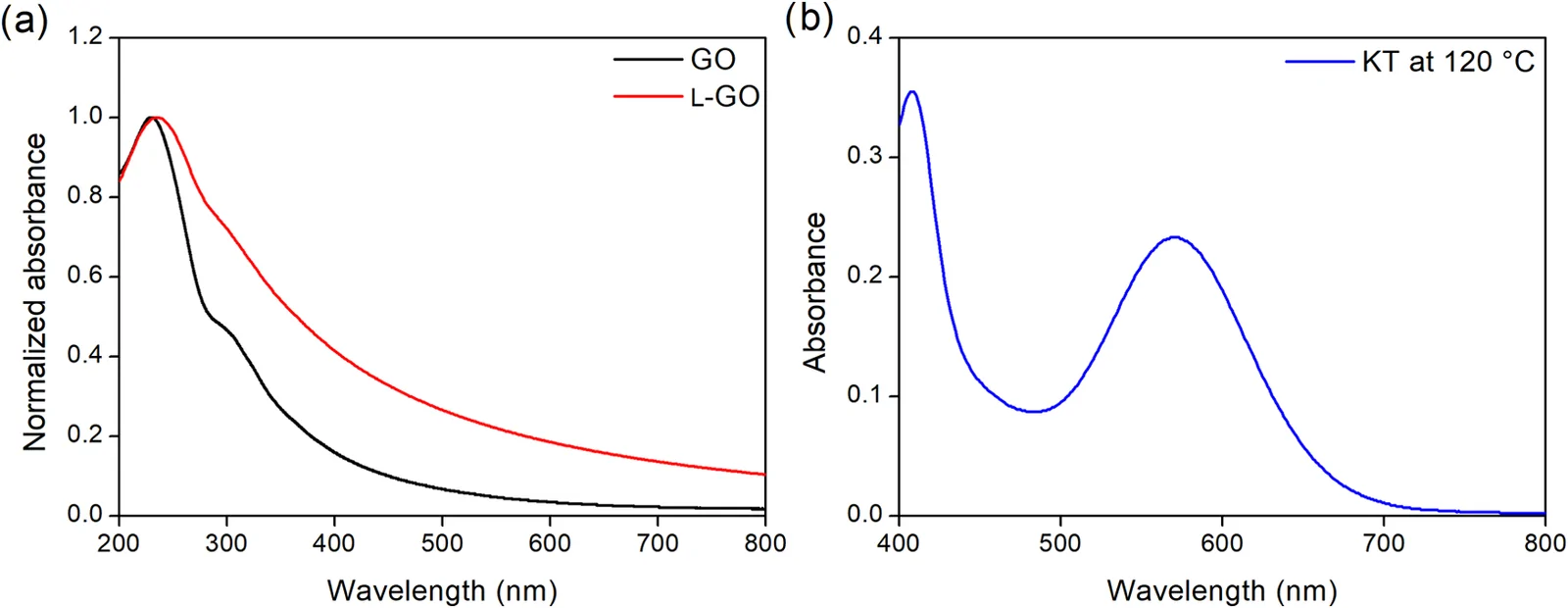
(a) UV–vis spectra of GO (black line) and l-GO (red line) recorded in water and at room temperature. Spectra are normalized with respect to the π–π* absorbance. (b) UV–vis spectrum of the colorimetric Kaiser test performed on l-GO at *T* = 120 °C.

To further investigate the reactivity of l-lysine with GO under alkaline conditions, Raman spectroscopy was performed. The spectrum of pristine GO (Figure 2, black line) displays the characteristic D band at ∼1350 cm^–1^, attributed to the defect-activated A_1g_ ring-breathing mode of sp^2^ carbon atoms, and the G band at ∼1605 cm^–1^, corresponding to the E_2g_ C=C stretching vibrations.^29,30^ Upon functionalization with l-lysine, the characteristic Raman signals are retained, while the *I*_D_/*I*_G_ ratio of the spectrum increases from 0.83 in GO to 0.91 in l-GO (Figure 2, red line). The increase in the Raman *I*_D_/*I*_G_ ratio is mainly attributed to base-induced disproportionation and structural rearrangement of GO, as confirmed by a control experiment conducted on a sample prepared in the absence of lysine (GO-NaOH, Figure S2). These processes generate additional defects or activate pre-existing ones, consequently enhancing the A_1g_ breathing mode.^26,30,31^ As to the Raman features of lysine,^32^ these are not visible, probably due to overlap with the dominant bands of GO.^33^

**Figure 2:**
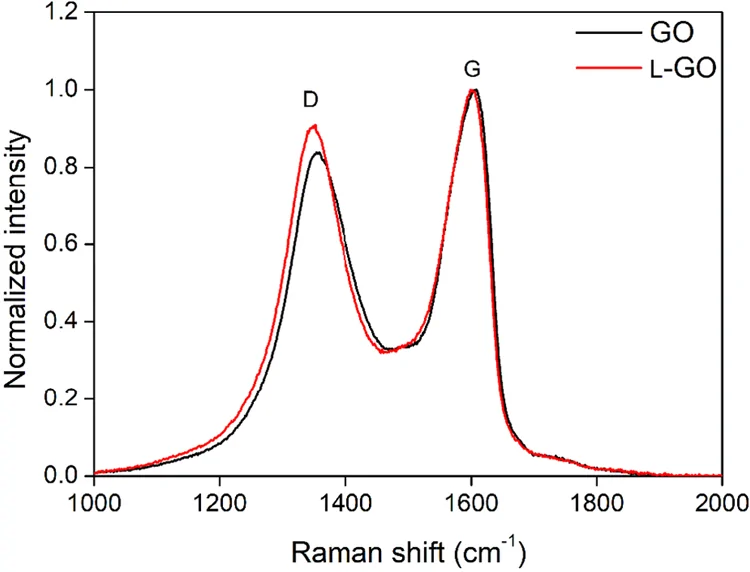
Raman spectra of GO (black line) and l-GO (red line) acquired using a 514 nm excitation wavelength laser source. Spectra are normalized with respect to the intensity of the G band.

The partial depletion of oxygen-based functionalities in GO suggested by Raman spectra after reaction with the amino acid was confirmed by cyclic voltammetry (CV) measurements (Figure S3). In fact, the reduction features attributed to epoxide and carbonyl groups at −0.92 V, as well as the signal at −1.24 V associated with hydroxyl groups,^34^ disappear upon reaction and merge into a featureless background.

In order to elucidate the chemical composition of l-GO and d-GO, XPS measurements were carried out. Analysis of the N 1s spectra confirms the successful incorporation of nitrogen-containing functionalities derived from the amino acids (Figures 3a and S4a). In both samples, the curve-fitting results (Table 1) reveal two distinct components, namely, a predominant peak at ∼400 eV attributed to amine groups (red), and a smaller contribution at ∼402 eV corresponding to protonated ammonium (−NH_3_^+^) species (blue).^35,36^ Using eq 4, the N% content was calculated to be 2.7% and 3.2% for l-GO and d-GO, respectively. These values correspond to an estimated functionalization degree of approximately 1.3% for the former and 1.6% for the latter, considering that each molecule of lysine contains two N atoms. The high-resolution C 1s XPS spectrum of reference GO (Figure 3b) can be deconvoluted into five distinct components located at binding energies of 284.8, 286.5, 287.0, 288.2, and 289.1 eV (Table 1). These peaks are respectively assigned to C=C and C=C–H sp^2^-hybridized carbon (pink), hydroxyl (cyan), epoxide (green), carbonyl (orange), and carboxyl (purple) functional groups.^34^ The resulting *R*_O/C_ atomic ratio of GO is 0.41, which correctly matches already reported data for GO.^31,34^ The C 1s spectra of l-GO (Figure 3c) and d-GO (Figure S4b) were fitted to the same type of components used for GO with an additional contribution at 285.7 eV needed to account for increased intensity in the 285–286 eV range, compatible with the freshly formed C–N chemical bonds.^31,37^ The intensity of this additional component, normalized to the number of C atoms (3) bound to N in the l-GO and d-GO samples, is 1.6 and 1.9%, respectively, which is fairly comparable to the above-mentioned functionalization degree.

**Figure 3:**
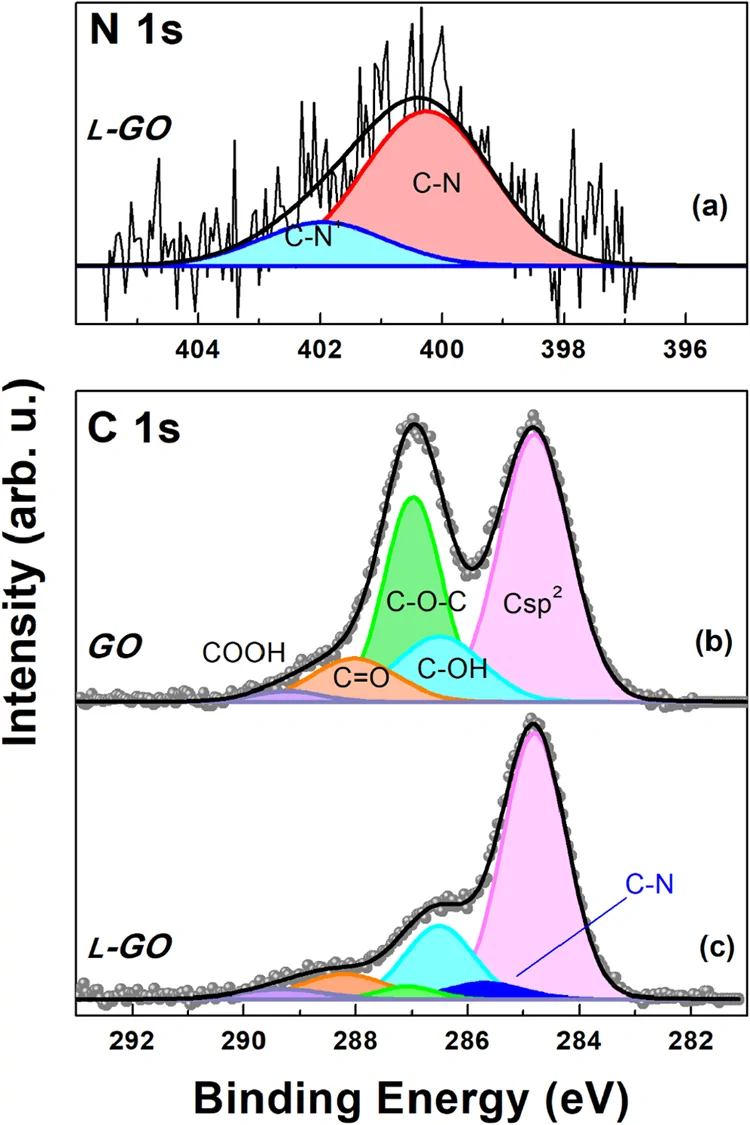
(a) High-resolution N 1s spectrum of l-GO. High-resolution C 1s spectra of (b) GO and (c) l-GO. Components of curve-fitting are reported as colored curves.

**Table 1:** BE Position and Percentage Amount (*A*%_FG_)­^a^ of Relevant Functional Groups Detected via C 1s and N 1s XPS Spectra^d^

C 1s BE position (eV)/*A*%_FG_ (%)								N 1s BE position (eV)		
Sample	C_sp2_	C–N	C–OH	COC	C=O	COOH	*R*_O/C_ ^b^	NH_2_	NH_3_^+^	N%^c^
GO	284.8/54.2	-	286.5/16.0	287.0/16.9	288.2/10.6	289.1/2.3	0.41			
L-GO	284.8/65.9	285.7/1.6	286.5/19.5	287.1/1.4	288.2/8.3	289.3/3.3	0.34	400.3	402.0	2.7
D-GO	284.8/66.1	285.7/1.9	286.5/21.8	287.0/1.8	288.2/5.1	289.1/3.3	0.33	400.0	401.7	3.2
GO-NaOH	284.8/56.4	-	286.5/16.3	286.9/13.5	288.0/11.5	289.2/2.2	0.40			

a Calculated with eq 1.

b Calculated with eq 2.

c Calculated with eq 4.

d N% is also reported.

Furthermore, the sample displays other significant changes in the relative peak areas (Figure 3c and Table 1). Specifically, the percentage of epoxide groups in the functionalized material decreases from 16.9% in pristine GO to 1.4% in l-GO and 1.8% in d-GO, as a consequence of the reactivity of the nucleophilic amine moieties of lysine, while the relative contribution of hydroxyl groups increases. Moreover, the sp^2^-carbon content in l-GO and d-GO rises from 54.2% in GO to 65.9 and 66.1%, respectively, while the resulting *R*_O/C_ values for l-GO and d-GO are 0.34 and 0.33, significantly lower than those of pristine GO (0.41). The results obtained from the GO-NaOH sample, prepared in the absence of lysine (Figure S5), show that in the sole presence of NaOH (pH = 10.5), GO reduction occurs to a minor yet significant extent, with an abatement of epoxide groups from 16.9 to 13.5% (Table 1), as also indicated by the Raman spectra. Therefore, it is apparent that lysine molecules under alkaline conditions manifest a considerable side-reactivity in terms of partial deoxygenation/reduction of GO, in addition to covalent grafting.

In order to investigate and clarify the mechanism of lysine grafting onto GO, the epoxide ring-opening reaction was modeled via DFT calculations involving the two possible lysine’s nucleophilic sites, *i*.*e*., the α- and ε-amino groups. This reaction in the presence of l-lysine was modeled simulating the GO layer with a molecular cluster of 54 carbon atoms (G54) with 6 epoxide groups in zigzag configuration in order to mimic the presence of oxygen-functional groups on the GO basal plane, and the approach of either the α-amine or the ε-amine side chain group to the GO surface was considered. The corresponding energy profiles are depicted in Figure 4.

**Figure 4:**
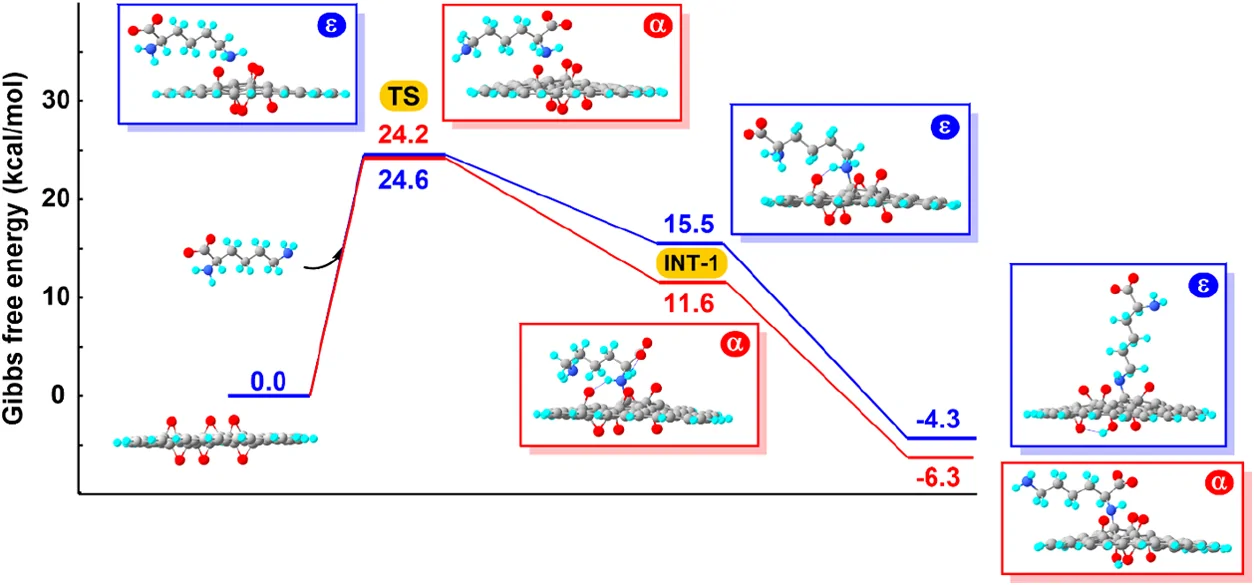
Energy profile of the epoxy-ring opening by l-lysine on the G54 carbon cluster. Red and blue data refer to the approach of the α- and ε-amine group to the GO surface, respectively.

The approach of the amino groups to the epoxy ring leads to the opening of the latter, thus forming the C–N bond. Intrinsic Reaction Coordinate (IRC) calculations of the proposed transition state (TS) are depicted in Figure S6. Once a charge-separated ammonium/alkoxide intermediate (INT-1) is generated, a rapid proton transfer assisted by the solvent (water) can occur, leading to the neutral product.^38^

The reaction energy profile shown in Figure 4 indicates that, under the experimental pH conditions, nucleophilic attack by the α-amino group is slightly favored over that by the ε-amino group, in good agreement with previous literature reports.^15,16,18^ Nevertheless, nucleophilic attack by the ε-amino group cannot be ruled out, since it is only slightly less favorable from both thermodynamic and kinetic perspectives. Further clarifications on how nucleophilicity influences the overall progress of the reaction path can be found in the Supporting Information material (Figure S7).

### Optical Activity of the Functionalized GO-Based Samples

Once the d- and l-GO samples were obtained, their chiral features were evaluated by CD spectroscopy. Despite the presence of grafted amino acids on the structure of GO, no CD signal was detected (Figure S8a). This feature is a consequence of both the strong absorption of GO below 250 nm, which masks the characteristic absorption of the amino acids, as well as the small amount of chiral amino acid covalently bound to GO after synthesis. In fact, solutions of pure l- and d-lysine did not return a detectable CD signal when present at the concentration determined via KT on d- and l-GO, whereas intense features were obtained for a 20 times higher concentration (Figure S8b). Importantly, the extensive purification via dialysis of the crude d- and l-GO products reduced any potential CD response from unreacted chiral lysine species (Figure S8c). Therefore, we opted to indirectly investigate the chirality of these samples by employing the d- and l-GO layers as chiral inducers for the formation of aggregates of the water-soluble TPPS molecules. In fact, under specific experimental conditions, TPPS forms specular and optically active J-aggregates, enabling the recording of high-ellipticity CD spectra.

Before examining the chirality induction exerted by the d- and l-GO samples on TPPS, we first carried out a systematic spectroscopic characterization of these species in the absence of graphene derivatives. The UV–vis spectrum of pure monomeric TPPS (1 μM) in water at its native pH of ∼7.9 shows a dominant Soret band at 413 nm, along with the Q bands, the most intense of which appears at 513 nm (Figure S9a, black line). Conversely, in the spectrum recorded at pH = ∼2.3 (Figure S9a, red line), because of the protonation of the pyrrole moieties of TPPS, both the Soret and Q bands are red-shifted from 413 to 435 nm and from 513 to 645 nm, respectively.

When the TPPS concentration exceeds 1 μM in acidic aqueous medium (0.1 M HCl) and 0.3 M NaCl is added in order to screen the inter-macrocycle repulsion, J-aggregates formation is initiated. This is evident in the UV–vis spectra (Figure S9b), with the appearance of a J-band at 491 nm within the Soret region, accompanied by a further red-shift of the Q-band at 708 nm and by the formation of one-dimensional nanorods, as proven by AFM images (Figure S10).^39–41^ The high value of ionic strength in the working conditions (NaCl 0.3 M) is expected to largely suppress Coulombic interactions in the solution. Although this may weaken the electrostatic interactions between functionalized GO and TPPS J-aggregates, we found that this salt concentration is essential for their formation (Figure S9), in agreement with previous experimental observations.^42^ Nonetheless, the interaction between d- and l-GO and TPPS, and the consequent chirality transfer at the macromolecular scale, can be expected to be carried on predominantly through dispersion forces. Once the optimal conditions for J-aggregates formation were found, their possible chiral induction exerted by the d- and l-GO samples was examined. Since GO layers strongly tend to aggregate in the presence of NaCl and easily precipitate upon centrifugation, only the corresponding supernatants (TPPS_sup (l-GO) and TPPS_sup (d-GO) samples) were collected and characterized. The UV–vis spectra of the two supernatants (Figure 5a, red and green traces) confirm the presence of J-aggregates, as evidenced by the J-band at 491 nm. However, a substantial fraction of monomeric TPPS is also detected—as indicated by the relative band intensities—likely because the J-aggregate species partially precipitate during centrifugation, or remain noncovalently bound to d- and l-GO. In line with this result, both supernatants enriched with monomeric TPPS display a fluorescence emission with a maximum at 668 nm upon excitation at 435 nm (Figure 5b, red and green traces), consistently with literature reports on pure TPPS,^43,44^ while the fluorescence from J-aggregates, excited at 490 nm, is mostly quenched and red-shifted to 713 nm (Figure 5b, blue and magenta traces). Fluorescence lifetime measurements on TPPS_sup (l-GO) and TPPS_sup (d-GO) revealed the presence of a fast decaying species (τ_fast_ = 0.22 ns) when the fluorescence is monitored at this wavelength (Figure S11), which is compatible with the J-aggregated form of TPPS (Figure S12).^45^

**Figure 5:**
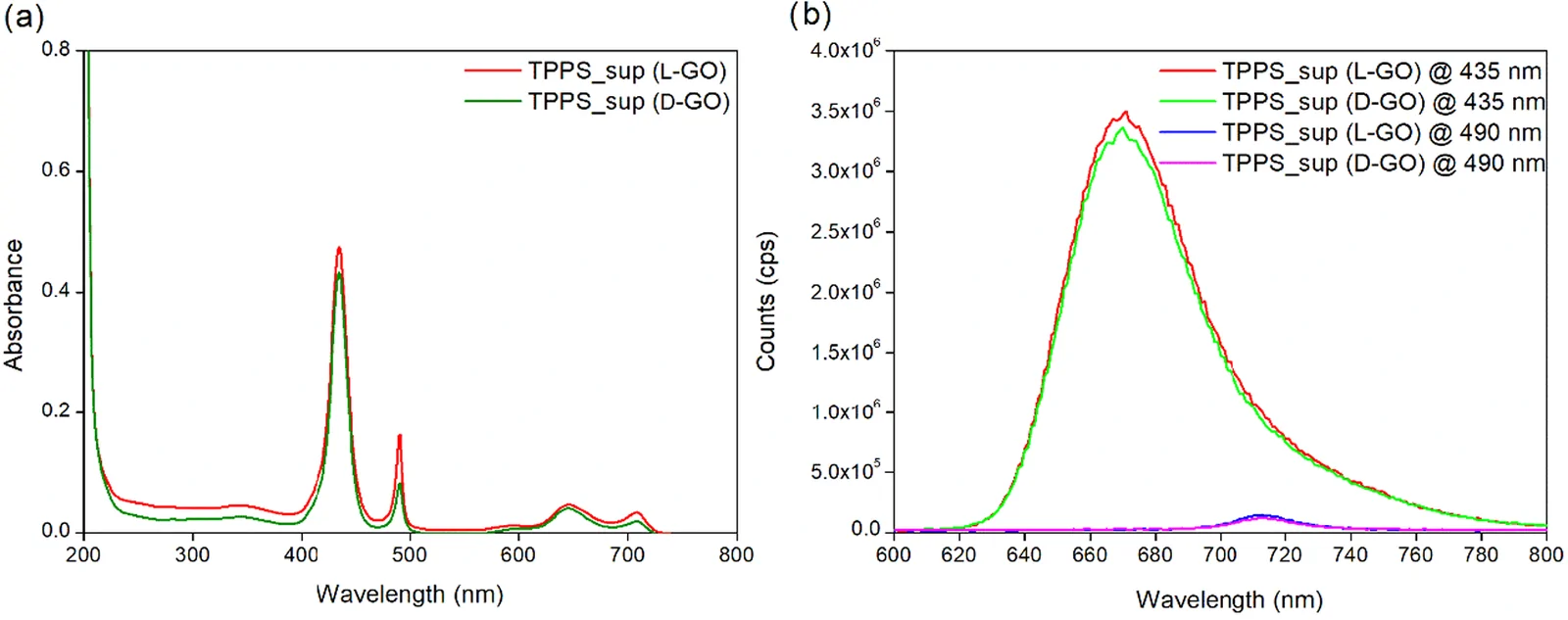
(a) UV–vis spectra recorded in water of TPPS_sup (l-GO) (red line) and TPPS_sup (d-GO) (green line) samples. (b) Fluorescence spectra recorded in water upon excitation at 435 and 490 nm of TPPS_sup (l-GO) (red and blue lines) and TPPS_sup (d-GO) (green and magenta lines) samples. Optical path length: 0.3 × 0.3 cm; *T* = 25 °C; for fluorescence spectra: slits 1.25/1.25 nm.

To investigate the chirality of the J-aggregates of TPPS, CD spectra were recorded for both supernatants using the spectra of the corresponding d- and l-GO aqueous dispersions as baselines (Figure S8a). The resulting spectra (Figure 6, red and green lines) display two bisignate CD bands with opposite Cotton effect centered at 489 and 492 nm, along with an inflection point and null ellipticity at 491 nm that coincide with the relative maximum of absorption (Figure 5a). In order to help the interpretation of these spectra, two control experiments were carried out, where GO and lysine were alternatively missing (Figure S13). In the former case (Figure S13a), d- and l-lysine were present as pure free species at the concentration determined via KT on d- and l-GO, and the resulting TPPS J-aggregate CD signal was fully compatible with that of Figure 6. Alternatively, in the absence of enantiomeric lysine molecules (Figure S13b), the sole GO solution was itself found to induce chirality to the J-aggregates of TPPS, but with a significantly different CD response, with only negative ellipticity. For a pure TPPS solution under the conditions for J-aggregation, the same kind of CD signal can be obtained (Figure S13b), which eventually points to a possible role of chiral impurities present in the water, yet ultrapure.^46^

**Figure 6:**
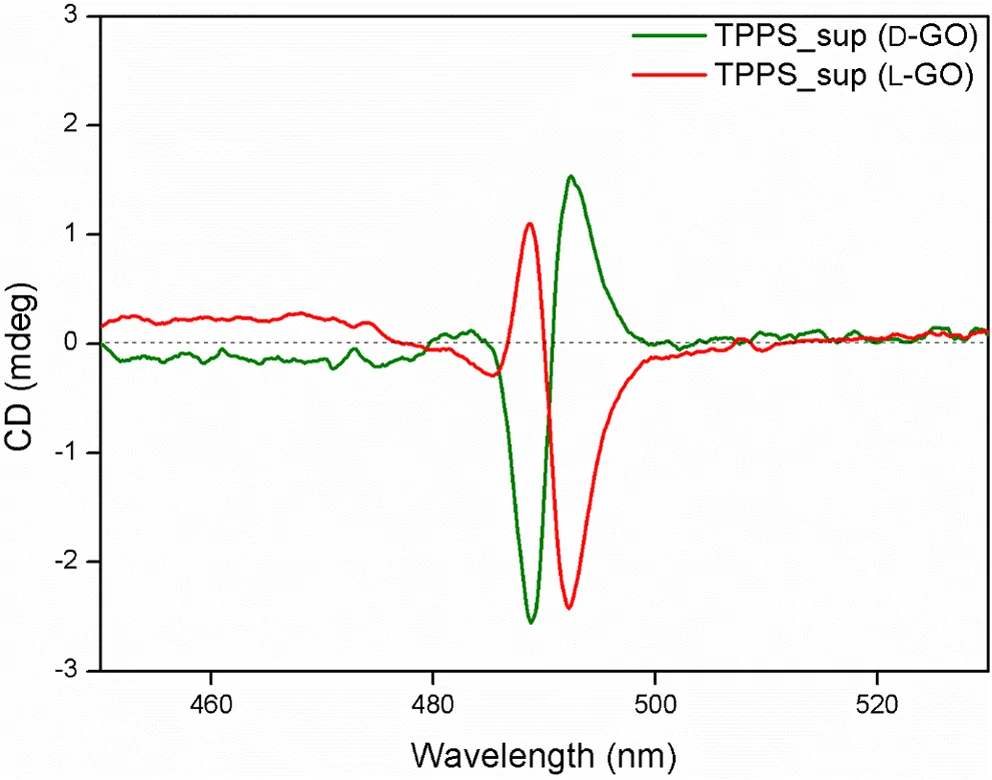
CD spectra recorded in water of TPPS_sup (l-GO) (red line) and TPPS_sup (d-GO) (green line) samples.

Therefore, the bisignate CD bands in Figure 6 associated with TPPS stacked in rod-like J-aggregates provide strong evidence of the transfer of chirality from the layers of GO functionalized with enantiomers of d- and l-lysine to TPPS, yielding enantiomeric J-aggregates.^21,47,48^ Notably, the chirality features of the d- and l-GO are preserved through time, with equivalent CD spectra obtained from newly formed TPPS_sup samples after months from the GO synthesis.

Once the chirality of the J-aggregates dispersed in the supernatants had been established, AFM measurements were carried out to examine their morphology. A few drops of the supernatant were deposited onto a mica substrate. The resulting AFM images (Figure 7) obtained from the TPPS_sup (l-GO) sample reveal one-dimensional, fiber-like TPPS J-aggregates. These structures exhibit micrometer-scale lengths and nanometric heights, ranging from ∼3 nm in the case of single fibers to more than ∼10 nm in the case of fully assembled fibers. Compared to J-aggregates formed in the absence of any GO derivative (Figure S10), here the fibers appear much longer and organized in thicker and more ordered strands. On the other hand, no significant differences in morphology or average dimensions were observed for the J-aggregates deriving from TPPS in the presence of d-GO (Figure S14), with no chiroptical information achievable from the comparison between the l- and d-derivatives.

**Figure 7:**
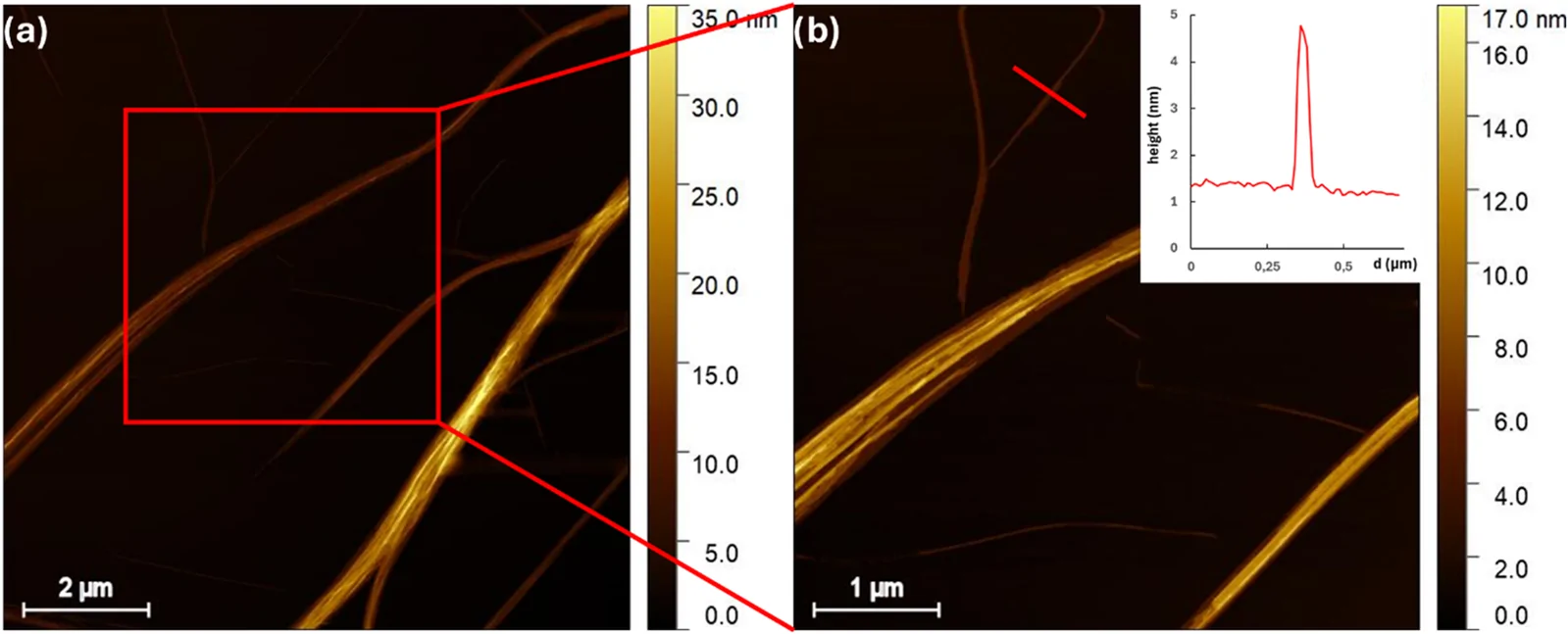
(a) Tapping mode AFM image (10 μm × 10 μm) and (b) its magnification (5 μm × 5 μm) from the J-aggregates of the TPPS_sup (l-GO) sample. In the inset, the height profile of a single fiber is shown.

## Conclusions

In this study, we have demonstrated a robust and simple strategy to impart chiral characteristics to GO through the covalent grafting of d- and l-lysine under mild alkaline conditions, which promote epoxide ring opening on the basal plane, involving both lysine’s aminic groups with a slight preference for that in the α-position. XPS and Raman characterization put in evidence that, aside from covalent grafting, lysine triggers a partial deoxygenation/reduction of GO, limiting the number of epoxide groups available for covalent bond formation. Since the direct recognition of the chirality of GO-bound lysine molecules was not possible due to their inherently low concentration, the chirally functionalized GO layers were used to induce the formation of chiral J-aggregates of a porphyrin probe, which proved to be highly sensitive to possible chiral contaminants as well. Owing to the rigorous dialysis-based purification protocol applied, it is reasonable that the chirality transfer could largely be attributed to the GO-bound chiral lysine centers.

Beyond establishing a reliable synthetic route, this work tackles a critical issue in the field, *i*.*e*., the effects of using an accurate purification protocol on the characterization results. In fact, our approach allows to limit the contribution of residual unbound chiral species to the bare minimum achievable, which makes it highly recommended for all future investigations in this field. The ability to engineer enantiomeric GO layers through covalent functionalization expands the design space of chirally decorated carbon-based nanomaterials. Furthermore, this lays the groundwork for their integration into asymmetric catalysis, chiral separations, and advanced chiroptical technologies.

## Methods

Reagents and solvents were purchased from Sigma-Aldrich, Fluka, Alfa Aesar, VWR, TCI, Fluorochem, and used as received, unless otherwise stated. Dialysis membranes with a cutoff of 12–14 kDa (Spectra/Por) were activated using Milli-Q water with a resistivity of 18.2 MΩ cm at 25 °C. 5,10,15,20-(Tetra-4-sulfonatophenyl)­porphyrin tetra-ammonium salt (TPPS—purity above 95%) and Kaiser test kit were provided by PorphyChem SAS and Sigma-Aldrich, respectively.

### Synthesis of Nanomaterials

Graphene Oxide (GO) was synthesized following our already reported procedure.^49–51^ l-Lysine-functionalized GO (l-GO) was prepared by dispersing 16.30 mg of GO in 30 mL of Milli-Q water. The dispersion was then sonicated for a few minutes at 30 °C to eliminate every GO-based lump, and then 32.60 mg of l-lysine were added. The native pH = ∼7.8 was corrected to ∼10.5 by adding a few aliquots of NaOH 0.5 mol L^–1^ dropwise, and subsequently, the aqueous dispersion was stirred at 40 °C for 72 h. The dispersion was then freeze-dried, and the solid material was resuspended in 5–6 mL of Milli-Q water to enable dialysis on a reduced sample volume. Dialysis was carried out in a 500 mL beaker for 3 days, with the external water replaced at least twice per day. The l-GO dialysate was then freeze-dried, obtaining ∼11 mg of a solid black powder. This powder tends to form dense aggregates that are difficult to disperse in water, even with sonication in a water bath. To prepare an aqueous dispersion of l-GO at a known concentration, the dialyzed sample was instead diluted to the mark in a 10 mL volumetric flask (Class A). A 5 mL aliquot of this dispersion was then freeze-dried and weighed. Based on the mass of the recovered solid, possibly including adventitious adsorbed water, the concentration of l-GO in the remaining 5 mL of dispersion was determined (1.05 mg mL^–1^). d-GO was prepared in the same manner but in the presence of d-lysine.

### Preparation of the J-Aggregates of Porphyrins

A volume of 625 μL of an aqueous dispersion of l-GO at a concentration of 1.05 mg mL^–1^ was added to 25 mL of an acidic water solution of 6 μM TPPS and 0.3 M NaCl (pH = 2.5—obtained by adding aliquots of 0.1 M HCl). Subsequently, the mixture was kept at rest for 72 h without stirring, and thereafter it was centrifuged at 8000 rpm × 30 min, yielding a precipitate and a supernatant. The latter was carefully collected using a pipet for further analysis. The same procedure was performed in the case of d-GO.

### Atomic Force Microscopy (AFM)

For the morphological investigation, the graphene-based samples were dispersed and diluted in Milli-Q water at a concentration of ∼0.001 mg mL^–1^, while the TPPS J-aggregates samples were obtained from their native solutions as prepared. Subsequently, a few drops of the related aqueous dispersions were deposited on mica and allowed to dry at room temperature. AFM measurements were performed in tapping mode on a Veeco AFM Multimode equipped with Nanoscope IIIa and using a PPP-NCSTR NANOSENSORS tip (Technical data: thickness 2.8 ± 1 μm, length 150 ± 10 μm, width 27 ± 7.5 μm, resonance frequency 76–263 kHz, force constant 1.2–29 N m^–1^, tip height 10–15 ± 1 μm, radius <7 nm). The images were recorded with a 512 × 512-pixel resolution and corrected by polynomial background filters using the software Gwyddion 2.56.

### Absorption and Fluorescence Spectroscopy

UV–vis absorption spectra were recorded with a Shimadzu UV-2600i Plus spectrophotometer at room temperature, employing quartz cuvettes with a 10 mm path length. Fluorescence spectra were recorded using a Horiba Jobin-Yvon spectrofluorometer (Fluoromax 2) at 25 °C (Peltier-thermostated sample holder from Quantum Northwest, Liberty Lake, WA, USA, controlled by the TC1 unit via the T-App software from the same manufacturer); slit width, cell path length, and long pass optical (LPO) filter are indicated in the Supporting Information. Fluorescence spectra were mathematically corrected for inner filter effect both at the excitation and emission wavelengths according to the following relationship^52^

$$F_{\mathrm{corr}}={F_{\mathrm{obs}}\times 10}^{\left(\mathrm{Abs@}\lambda_{\mathrm{exct}}+\mathrm{Abs@}\lambda_{\mathrm{em}}\right)/2}$$

where *F*_corr_ and *F*_obs_ are the corrected and measured fluorescence, respectively; Abs@λ_exct_ is the optical density of the sample at the excitation wavelength; and Abs@λ_em_ is a range of optical density values shown by the sample at the wavelengths acquired in the fluorescence emission spectra. Fluorescence lifetime measurements were performed at 25 °C on a home-built instrument described elsewhere.^53,54^ Fluorescence lifetime data were analyzed with the global fluorescence decay data analysis software FluoFit (Ver. 4.6 Pro; PicoQuant GmbH, Germany). Details on the fitting procedure adopted for the different samples are provided in the Supporting Information.

### Circular Dichroism (CD) Spectroscopy

CD spectra were obtained with a JASCO J-715 spectropolarimeter equipped with a Peltier device for temperature control using quartz cells of 1 cm. Measurements were run in the 500–200 nm spectral range at constant temperature and with an instrument scanning speed of 100 nm min^–1^, with a response time of 1 s and a bandwidth of 1 nm. Spectra are reported in millidegrees (mdeg).

### Raman Spectroscopy

Raman spectra were acquired at room temperature in backscattering geometry with an inVia Renishaw micro-Raman spectrometer equipped with an air-cooled CCD detector and super-Notch filters. An Ar^+^ ion laser (λ_laser_ = 514 nm) was used, coupled to a Leica DLML microscope with a 20× objective. The resolution was set to 2 cm^–1^, and the spectra were calibrated using the 520.5 cm^–1^ line of a silicon wafer. Raman spectra were acquired in several (6–10) different spots on the surface of the samples. Each spectrum was acquired with 50% of power, 10 s of spectral acquisition, and 20 scans.

### Electrochemical Measurements

Electrochemical characterization was conducted by drop-casting 50 μL of aqueous dispersions of GO and GO-based samples at a concentration of 0.06 mg mL^–1^ onto H-terminated n-doped Si(100) surfaces (Siegert Wafer, N-doped with a resistivity <0.005 Ω cm) with a thickness of 525 ± 25 μm and allowed to dry at room temperature. These samples were used as working electrodes in an electrochemical cell, where the electrode window area was roughly the same as the electroactive drop-casted GO deposit (∼1 cm^2^). The electrolyte used was an aqueous 0.1 M phosphate (K_2_HPO_4_/KH_2_PO_4_) buffered saline (PBS) solution, with pH = 7.2, and the electrodes were a Ag/AgCl (sat., reference, *E* = 0.198 V vs. NHE, all potential values will henceforth be referred to this electrode) and a platinum wire (counter electrode). The samples were studied by means of cyclic voltammetry (CV), scanning the potential between open-circuit potential (OCP, 0.06 V) and −1.55 V, with a potential scan rate of 20 mV s^–1^ for 3–4 cycles. The choice of this potential scan rate represents a good compromise between intensity and resolution of the recorded reduction features. After electrochemical investigations, samples were extracted from the cell and thoroughly rinsed with distilled water. All electrochemical measurements were performed using a Bio-Logic SP-150 potentiostat/galvanostat driven by the Bio-Logic EC-Lab software.

### X-ray Photoelectron Spectroscopy (XPS)

A freshly prepared H-terminated Si(100) surface was used as a support for the drop-casting of aqueous dispersions of GO, d-, and l-GO. XPS measurements were carried out using an Omicron NanoTechnology (Uppsala, Sweden) Multiprobe MXPS system equipped with a monochromatic Al Kα (*h*ν = 1486.7 eV) X-ray source (Scienta Omicron XM-1000), with 14 kV accelerating voltage and 16 mA emission current. The C 1s, N 1s, and O 1s photoionization regions were acquired using an analyzer pass energy of 20 eV. A takeoff angle of 21° with respect to the sample surface normal was adopted. The experimental spectra were theoretically reconstructed by fitting the secondary electrons background to a Shirley function and the elastic peaks to pseudo-Voigt functions with a 70:30 Gaussian–Lorentzian ratio.

The relative percentage amount of the different functional groups (FGs) of GO (*A*%_FG_) was determined through the area of the FG peaks (*A*_FG_) within the curve-fitting envelope of the C 1s region (epoxy and C–N area must be divided by 2 and 3, respectively), according to the following equation^55^

1 $${A\%}_{\mathrm{FG}}=\frac{A_{\mathrm{FG}}}{A_{C=C}+A_{C\text{−}N}+A_{C-\mathrm{OH}}+1/2A_{C\text{−}O\text{−}C}+A_{C=O}+A_{\mathrm{COOH}}}\times 100$$

The oxygen content was determined through the *R*_O/C_ ratio obtained after the curve-fitting of the C 1s region, using the following equation^56^

$$R_{O/C}=\frac{A_{C\text{−}\mathrm{OH}}+1/2A_{C\text{−}O\text{−}C}+A_{C=O}+2A_{\mathrm{COOH}}}{A_{C=C}+A_{C-N}+A_{C-\mathrm{OH}}+A_{C-O\text{−}C}+A_{C=O}+A_{\mathrm{COOH}}}$$ 2

This approach rules out any possible contributions to the oxygen content deriving either from contaminants or from the support material (Si).

The nitrogen percentage with respect to carbon (N%) was calculated using the intensity (*I*_*i*_) of the N 1s and C 1s regions, considering normalization of the spectral area (*A*_*i*_) with respect to the square root of photoelectron kinetic energy (KE_*i*_) and the relative sensitivity factor (RSF_*i*_), according to the following equations

3 $$I_{i}=\frac{A_{i}}{\sqrt{KE_{i}}\cdot RSF_{i}}$$

4 $$N\%=\frac{I_{N_{1s}}}{I_{N_{1s}}+I_{C_{1s}}}\times 100$$

According to the Al Kα photons used, the KE values are 1202 eV for C 1s and 1086 eV for N 1s photoelectrons, while the corresponding RSFs are 1 and 1.8, respectively.^57^

### Computational Details

Geometry optimizations of all reactants, products, and transition states were achieved along the entire investigated reaction. Calculations were performed adopting the M06 hybrid meta-GGA functional^58,59^ with the standard all-electron 6–311+G** basis set.^60^ Molecular geometry optimization of stationary points was obtained without symmetry constraints and using analytical gradient techniques. The transition states were proven by the presence of a single imaginary frequency. Frequency analysis was performed to obtain thermochemical information about the reaction pathways at 298 K upon the harmonic approximation. Solvation contribution to the energy profile was evaluated within the Polarizable Continuum Model (PCM) approach using water as a solvent. All of the calculations were performed with a full optimization and frequency calculation with the less demanding 6–311G**, followed by a single point in the presence of diffuse function (6–311+G**).^61^ Then, the Gibbs free energy reaction was obtained using eq 5

$${\Delta G}_{6\text{−}311+G**}=\Delta \mathrm{SC}F_{6\text{−}311+G**}+\left({\Delta G}_{6\text{−}311G**}-\Delta \mathrm{SC}F_{6\text{−}311G**}\right)$$ 5

All calculations were carried out using Gaussian 16 (G16) software.^62^

## Supplementary Material



## Notes

AFM images of pristine GO and J-aggregates of pure TPPS and TPPS_sup (d-GO); cyclic voltammetry of pristine GO and l-GO; Raman spectrum of GO-NaOH; XPS spectra of d-GO and GO-NaOH; DFT calculations regarding mechanistic studies and NBO analysis; UV–vis spectra of TPPS solutions; CD spectra of control samples; fluorescence emission and lifetime measurements of TPPS and TPPS_sup samples (PDF)

## References

[1] Wang G., Qu A., Sun M., Xu J., Kuang H. (2024). Chemical Mechanisms
and Biological Effects of Chiral Nanomaterials. Acc. Mater. Res..

[2] Ma B., Bianco A. (2023). Regulation of biological
processes by intrinsically
chiral engineered materials. Nat. Rev. Mater..

[3] Niu X., Zhao R., Yan S., Pang Z., Li H., Yang X., Wang K. (2023). Chiral Materials:
Progress, Applications,
and Prospects. Small.

[4] Di
Noja S., Amato F., Zinna F., Di Bari L., Ragazzon G., Prato M. (2022). Transfer of Axial Chirality to the Nanoscale Endows Carbon Nanodots
with Circularly Polarized Luminescence. Angew.
Chem., Int. Ed..

[5] Nitti A., Pasini D. (2020). Aggregation-Induced Circularly Polarized Luminescence
Chiral Organic Materials for Emerging Optical Technologies. Adv. Mater..

[6] Probst P. T., Dong Y., Zhou Z., Aftenieva O., Fery A. (2024). Bottom-Up Assembly of Inorganic Particle-Based
Chiroptical Materials. Adv. Opt. Mater..

[7] Qi H., Hegmann T. (2008). Postsynthesis racemization and place exchange reactions.
another step to unravel the origin of chirality for chiral ligand-capped
gold nanoparticles. J. Am. Chem. Soc..

[8] Suzuki N., Wang Y., Elvati P., Qu Z. B., Kim K., Jiang S., Baumeister E., Lee J., Yeom B., Bahng J. H. (2016). Chiral Graphene Quantum
Dots. ACS Nano.

[9] Brisebois P. P., Siaj M. (2020). Harvesting graphene
oxide-years 1859 to 2019: A review of its structure,
synthesis, properties and exfoliation. J. Mater.
Chem. C.

[10] Zhao B., Yang S., Deng J., Pan K. (2021). Chiral Graphene Hybrid
Materials: Structures, Properties, and Chiral Applications. Adv. Sci..

[11] Cheng Q., Pei H., Ma Q., Guo R., Liu N., Mo Z. (2023). Chiral graphene
materials for enantiomer separation. Chem. Eng.
J..

[12] Niu X., Liu Y., Zhao R., Wang L., Yuan M., Zhao H., Li H., Yang X., Wang K. (2024). Chiral carbon
nanostructures: a gateway
to promising chiral materials. J. Mater. Chem.
A.

[13] Qie F., Guo J., Tu B., Zhao X., Zhang Y., Yan Y. (2018). β-Cyclodextrin
Functionalized Nanoporous Graphene Oxides for Efficient Resolution
of Asparagine Enantiomers. Chem. - Asian J..

[14] Meng C., Sheng Y., Chen Q., Tan H., Liu H. (2017). Exceptional
chiral separation of amino acid modified graphene oxide membranes
with high-flux. J. Membr. Sci..

[15] Mantovani S., Marforio T. D., Khaliha S., Pintus A., Kovtun A., Tunioli F., Favaretto L., Bianchi A., Navacchia M. L., Palermo V. (2023). Amino
acid-driven adsorption of emerging contaminants
in water by modified graphene oxide nanosheets. Environ. Sci. Water Res. Technol..

[16] Moro G., Khaliha S., Pintus A., Mantovani S., Feltracco M., Gambaro A., Marforio T. D., Calvaresi M., Palermo V., Melucci M., Zanardi C. (2024). Amino acid
modified
graphene oxide for the simultaneous capture and electrochemical detection
of glyphosate. Mater. Today Chem..

[17] Liu J., Yuan W., Li C., Cheng M., Su Y., Xu L., Chu T., Hou S. (2021). L -Cysteine-Modified Graphene Oxide-Based
Membrane for Chiral Selective Separation. ACS
Appl. Mater. Interfaces.

[18] Mantovani S., Khaliha S., Marforio T. D., Kovtun A., Favaretto L., Tunioli F., Bianchi A., Petrone G., Liscio A., Palermo V. (2022). Facile
high-yield synthesis and purification of lysine-modified
graphene oxide for enhanced drinking water purification. Chem. Commun..

[19] Mo Z., Gou H., He J., Yang P., Feng C., Guo R. (2012). Controllable
synthesis of functional nanocomposites: Covalently functionalize graphene
sheets with biocompatible L-lysine. Appl. Surf.
Sci..

[20] Huang H., Hu L., Sun Y., Liu Y., Kang Z., MacFarlane D. R. (2019). Preparation
of chiral graphene oxides by covalent attachment of chiral cysteines
for voltammetric recognition of tartrates. Microchim.
Acta.

[21] Thorpe S. L., Snyder G. N., Mammana A. (2020). Spectroscopic study
of porphyrin
self-assembly: Role of pH, time, and chiral template. Chirality.

[22] Zhang L., Wang T., Jiang J., Liu M. (2023). Chiral porphyrin assemblies. Aggregate.

[23] Gacka E., Wojcik A., Mazurkiewicz-Pawlicka M., Malolepszy A., Stobiński L., Kubas A., Hug G. L., Marciniak B., Lewandowska-Andralojc A. (2019). Noncovalent Porphyrin-Graphene Oxide
Nanohybrids: The pH-Dependent Behavior. J. Phys.
Chem. C.

[24] Shim S. (2019). Acid–Base
Chemistry of Porphyrin/Graphene Oxide Complex: Role of Electrostatic
Interaction. Bull. Korean Chem. Soc..

[25] Koniev O., Wagner A. (2015). Developments and recent
advancements in the field of
endogenous amino acid selective bond forming reactions for bioconjugation. Chem. Soc. Rev..

[26] Dimiev A. M., Alemany L. B., Tour J. M. (2013). Graphene
oxide. Origin of acidity,
its instability in water, and a new dynamic structural model. ACS Nano.

[27] Filippini G., Amato F., Rosso C., Ragazzon G., Vega-Peñaloza A., Companyó X., Dell’Amico L., Bonchio M., Prato M. (2020). Mapping the
Surface Groups of Amine-Rich Carbon Dots Enables Covalent Catalysis
in Aqueous Media. Chem.

[28] Gentile G., Mamone M., Rosso C., Amato F., Lanfrit C., Filippini G., Prato M. (2023). Tailoring the Chemical Structure
of Nitrogen-Doped Carbon Dots for Nano-Aminocatalysis in Aqueous Media. ChemSusChem.

[29] Guo S., Garaj S., Bianco A., Ménard-Moyon C. (2022). Controlling
covalent chemistry on graphene oxide. Nat. Rev.
Phys..

[30] Dimiev A. M., Halbig C. E., Talyzin A. (2026). A Critical Review to
Avoid Common
Misinterpretations in Characterizing Graphene Oxide. Chem. Rev..

[31] Amato F., Possenti D., Daino M., Giaccari L., Marino R., Lucianatelli M., Motta A., Olivo G., Marrani A. G. (2026). Iron-mediated
extensive amination of graphene-based nanomaterials. Carbon.

[32] Gorelik V. S., Sverbil V. P., Gorshunov B. P., Seredin A. I. (2017). Pseudoscalar lattice
modes in the amino acid crystals and DNA. J.
Phys. Conf. Ser..

[33] Arslan B. Z., Yurtsever M. (2026). IR and Raman
spectroscopy reveal amino acid–surface
interactions on B- and N-doped hydroxylated graphene quantum dots:
a DFT study. RSC Adv..

[34] Ferrari I., Motta A., Zanoni R., Scaramuzzo F. A., Amato F., Dalchiele E. A., Marrani A. G. (2023). Understanding the
nature of graphene oxide functional groups by modulation of the electrochemical
reduction: A combined experimental and theoretical approach. Carbon.

[35] Polzonetti G., Battocchio C., Dettin M., Gambaretto R., Di Bello C., Carravetta V., Monti S., Iucci G. (2008). Self-assembling
peptides: A combined XPS and NEXAFS investigation on the structure
of two dipeptides Ala-Glu, Ala-Lys. Mater. Sci.
Eng., C.

[36] Beamson, G. ; Briggs, D. High Resolution XPS of Organic Polymers, The Scienta ESCA300 Database; John Wiley & Sons Ltd.: Chichester, 1992.

[37] Niedziałkowski P., Ossowski T., Zięba P., Cirocka A., Rochowski P., Pogorzelski S. J., Ryl J., Sobaszek M., Bogdanowicz R. (2015). Poly-l-lysine-modified
boron-doped diamond electrodes for the amperometric detection of nucleic
acid bases. J. Electroanal. Chem..

[38] Sierra U., Cuara E., Mercado A., Díaz-Barriga E., Bahena A., Cortés A., Martínez J. P., Solà M., Fernández S. (2021). Efficient
synthesis of amine-functionalized
graphene oxide by ultrasound-assisted reactions and density functional
theory mechanistic insight. Appl. Nanosci..

[39] Friesen B. A., Nishida K. R. A., McHale J. L., Mazur U. (2009). New nanoscale insights
into the internal structure of tetrakis­(4- sulfonatophenyl) porphyrin
nanorods. J. Phys. Chem. C.

[40] Schwab A. D., Smith D. E., Rich C. S., Young E. R., Smith W. F., De Paula J. C. (2003). Porphyrin nanorods. J. Phys.
Chem. B.

[41] Gaeta M., Randazzo R., Costa C., Purrello R., D’Urso A. (2023). Enantiomeric
Resolution and Enantiomer Isolation of H2TPPS4 J-Aggregate from Aqueous
Solution Is Enabled by Vortexes. Chem. - Eur.
J..

[42] Andrade S. M., Teixeira R., Costa S. M. B., Sobral A. J. F. N. (2008). Self-aggregation
of free base porphyrins in aqueous solution and in DMPC vesicles. Biophys. Chem..

[43] Maiti N. C., Ravikanth M., Mazumdar S., Periasamy N. (1995). Fluorescence
dynamics of noncovalently linked porphyrin dimers and aggregates. J. Phys. Chem. A.

[44] Van
Patten P. G., Shreve A. P., Donohoe R. J. (2000). Structural and photophysical
properties of a water-soluble porphyrin associated with polycations
in solution and electrostatically-assembled ultrathin films. J. Phys. Chem. B.

[45] Maiti N. C., Mazumdar S., Periasamy N. (1998). J- and H-Aggregates of Porphyrin–Surfactant
Complexes: Time-Resolved Fluorescence and Other Spectroscopic Studies. J. Phys. Chem. B.

[46] El-Hachemi Z., Escudero C., Arteaga O., Canillas A., Crusats J., Mancini G., Purrello R., Sorrenti A., D’urso A., Ribo J. M. (2009). Chiral sign selection
on the J-aggregates of diprotonated
tetrakis-(4-sulfonatophenyl)­porphyrin by traces of unidentified chiral
contaminants present in the ultra-pure water used as solvent. Chirality.

[47] Zeng L., He Y., Dai Z., Wang J., Cao Q., Zhang Y. (2009). Chiral induction,
memory, and amplification in porphyrin homoaggregates based on electrostatic
interactions. ChemPhysChem.

[48] Zhang L., Liu M. (2009). Supramolecular chirality
and chiral inversion of tetraphenylsulfonato
porphyrin assemblies on optically active polylysine. J. Phys. Chem. B.

[49] Amato F., Motta A., Giaccari L., Di Pasquale R., Scaramuzzo F. A., Zanoni R., Marrani A. G. (2023). One-pot
carboxyl
enrichment fosters water-dispersibility of reduced graphene oxide:
a combined experimental and theoretical assessment. Nanoscale Adv..

[50] Amato F., Perini G., Friggeri G., Augello A., Motta A., Giaccari L., Zanoni R., De Spirito M., Palmieri V., Marrani A. G., Papi M. (2023). Unlocking the Stability
of Reduced Graphene Oxide Nanosheets in Biological Media via Use of
Sodium Ascorbate. Adv. Mater. Interfaces.

[51] Tiberio F., Amato F., Desiderio C., Vincenzoni F., Perini G., Moretti I., Augello A., Friggeri G., Cui L., Giaccari L. (2024). The osteoconductive properties of graphene-based
material surfaces are finely tuned by the conditioning layer and surface
chemistry. Mater. Adv..

[52] Lakowicz, J. R. Principles of Fluorescence Spectroscopy, 3rd ed.; Springer Science+Business Media: New York, USA, 2006.

[53] Airoldi M., Barone G., Gennaro G., Giuliani A. M., Giustini M. (2014). Interaction
of Doxorubicin with Polynucleotides. A Spectroscopic Study. Biochemistry.

[54] Tasca E., Del Giudice A., Galantini L., Schillén K., Giuliani A. M., Giustini M. (2019). A fluorescence
study of the loading
and time stability of doxorubicin in sodium cholate/PEO-PPO-PEO triblock
copolymer mixed micelles. J. Colloid Interface
Sci..

[55] Amato F., Fazi M., Giaccari L., Colecchia S., Perini G., Palmieri V., Papi M., Altimari P., Motta A., Giustini M. (2025). Isolation
by dialysis
and characterization of luminescent oxidized carbon nanoparticles
from graphene oxide dispersions: a facile novel route towards a more
controlled and homogeneous substrate with a wider applicability. Nanotechnology.

[56] Marrani A. G., Motta A., Palmieri V., Perini G., Papi M., Dalchiele E. A., Schrebler R., Zanoni R. (2020). A comparative experimental
and theoretical study of the mechanism of graphene oxide mild reduction
by ascorbic acid and: N -acetyl cysteine for biomedical applications. Mater. Adv..

[57] Scofield J. H. (1976). Hartree-Slater
subshell photoionization cross-sections at 1254 and 1487 eV. J. Electron Spectrosc. Relat. Phenom..

[58] Mardirossian N., Head-Gordon M. (2017). Thirty years
of density functional theory in computational
chemistry: An overview and extensive assessment of 200 density functionals. Mol. Phys..

[59] Zhao Y., Truhlar D. G. (2008). The M06 suite of
density functionals for main group
thermochemistry, thermochemical kinetics, noncovalent interactions,
excited states, and transition elements: two new functionals and systematic
testing of four M06-class functionals and 12 other function. Theor. Chem. Acc..

[60] McLean A. D., Chandler G. S. (1980). Contracted Gaussian
basis sets for molecular calculations.
I. Second row atoms, Z = 11–18. J. Chem.
Phys..

[61] Bursch M., Mewes J.-M., Hansen A., Grimme S. (2022). Best-Practice DFT Protocols
for Basic Molecular Computational Chemistry. Angew. Chem., Int. Ed..

[62] Frisch, M. J. ; Trucks, G. W. ; Schlegel, H. B. ; Scuseria, G. E. ; Robb, M. A. ; Cheeseman, J. R. ; Scalmani, G. ; Barone, V. ; Petersson, G. A. ; Nakatsuji, H. Gaussian 16 Revision B.01; Gaussian Inc. 2016.

