# Supplementary material for: Introducing Chirality into Graphene Oxide Layers through a Facile Amino Acid-Based Covalent Functionalization
Source: ACS Omega. 2026 Oct 26;11(44):68696–705. doi: 10.1021/acsomega.6c07448 (PMC13756310; doi:10.1021/acsomega.6c07448)
Supplement: Supplementary file 1 [file ao6c07448_si_001.pdf]

Introducing Chirality into Graphene Oxide Layers through a Facile Amino Acid-Based Covalent Functionalization

Francesco Amato,[1,2]‡ Samuele De Santis,[1]‡ Matteo Daino,[1] Leonardo Giaccari,[1] Alessandro Motta,[1,3] Mauro Giustini,[1] Francesca Anna Scaramuzzo,[4] Anita Scipioni,[1] Andrea Giacomo Marrani[1*]*

[1]Dipartimento di Chimica, Sapienza Università di Roma, p.le A. Moro 5, I-00185 Rome, Italy

[2]Department for Sustainability, ENEA, Casaccia Research Centre, I-00123 Rome, Italy

[3]INSTM Consortium, UdR Roma, P.le A. Moro 5, I-00185, Rome, Italy

[4]Dipartimento di Scienze di Base e Applicate per l'Ingegneria (S.B.A.I.), Sapienza Università di Roma, Via del Castro Laurenziano 7, I-00161, Rome, Italy

Supporting Information

**Table S1.** Relevant information on other published works referenced in the main text reporting chiral amino acids bound to GO and their functionalization degree as obtained from XPS.				
**Chirality inducer**	**Functionalization Reaction**	**Purification method**	**N% or N/C via XPS**	**Ref. in main text**
L-Glu	Amide coupling	Dialysis	N/C 0.1	14
L-Glu; L-Met methyl ester	Epoxide ring-opening (microwave assisted with basic AA solution)	Microfiltration	GO-Met N/C 0.011; GO-Glu N/C 0.009; GO-Lys N/C 0.04	15
L-Arg, L-Met, L-Lys	Epoxide ring-opening (basic pH)	Microfiltration	NA	16
L-Cys	Thiol–ene click method	Dialysis	1.19 N%	17
L-Lys	Epoxide ring-opening (microwave assisted with basic AA solution)	Microfiltration	3.2 N%	18
Lys-Cu-Lys	Epoxide ring-opening	Centrifugation	NA	19
L- and D-Cys	One-step hydrothermal method	water washing	11.6 and 11.2 N%	20

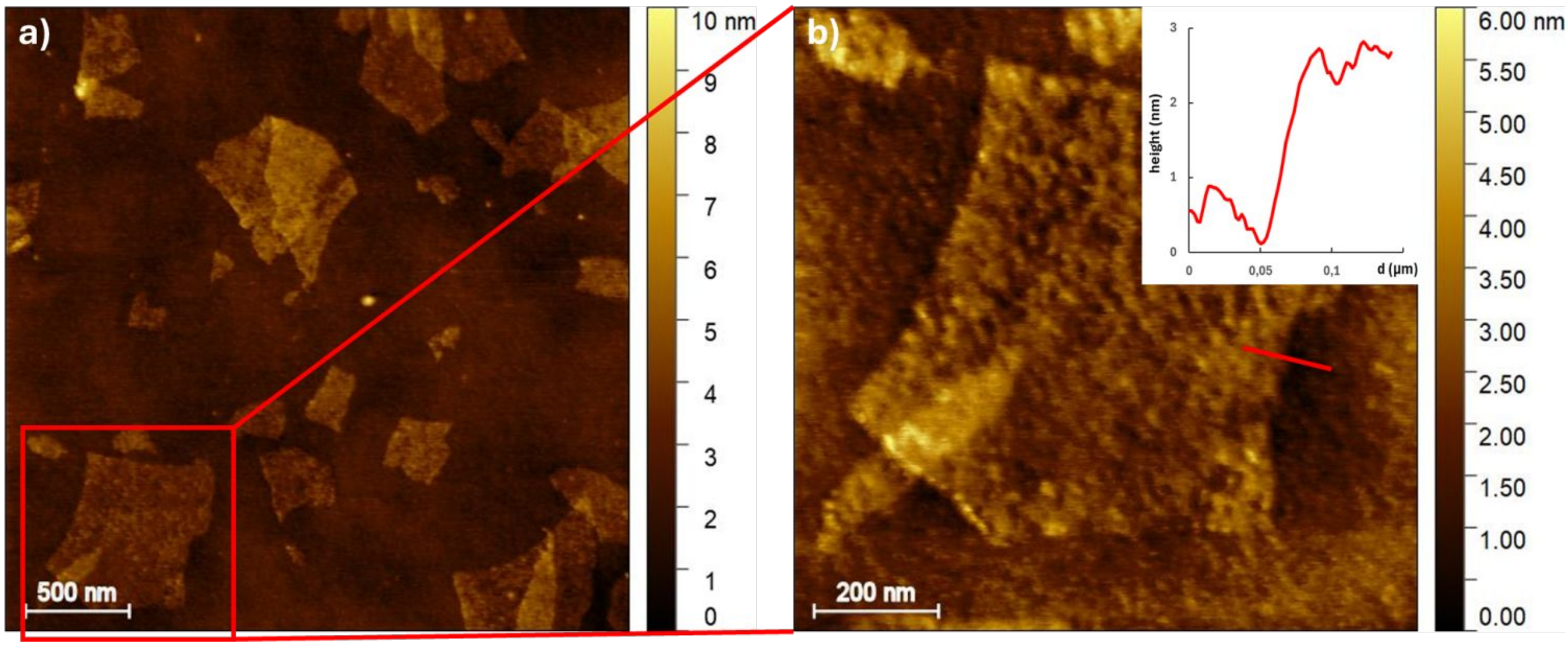

Figure S1. Tapping mode AFM images of GO: (a) 3.5 µm × 3.5 µm, (b) 1 µm × 1 µm. Inset: height profile corresponding to the red bar in (b).

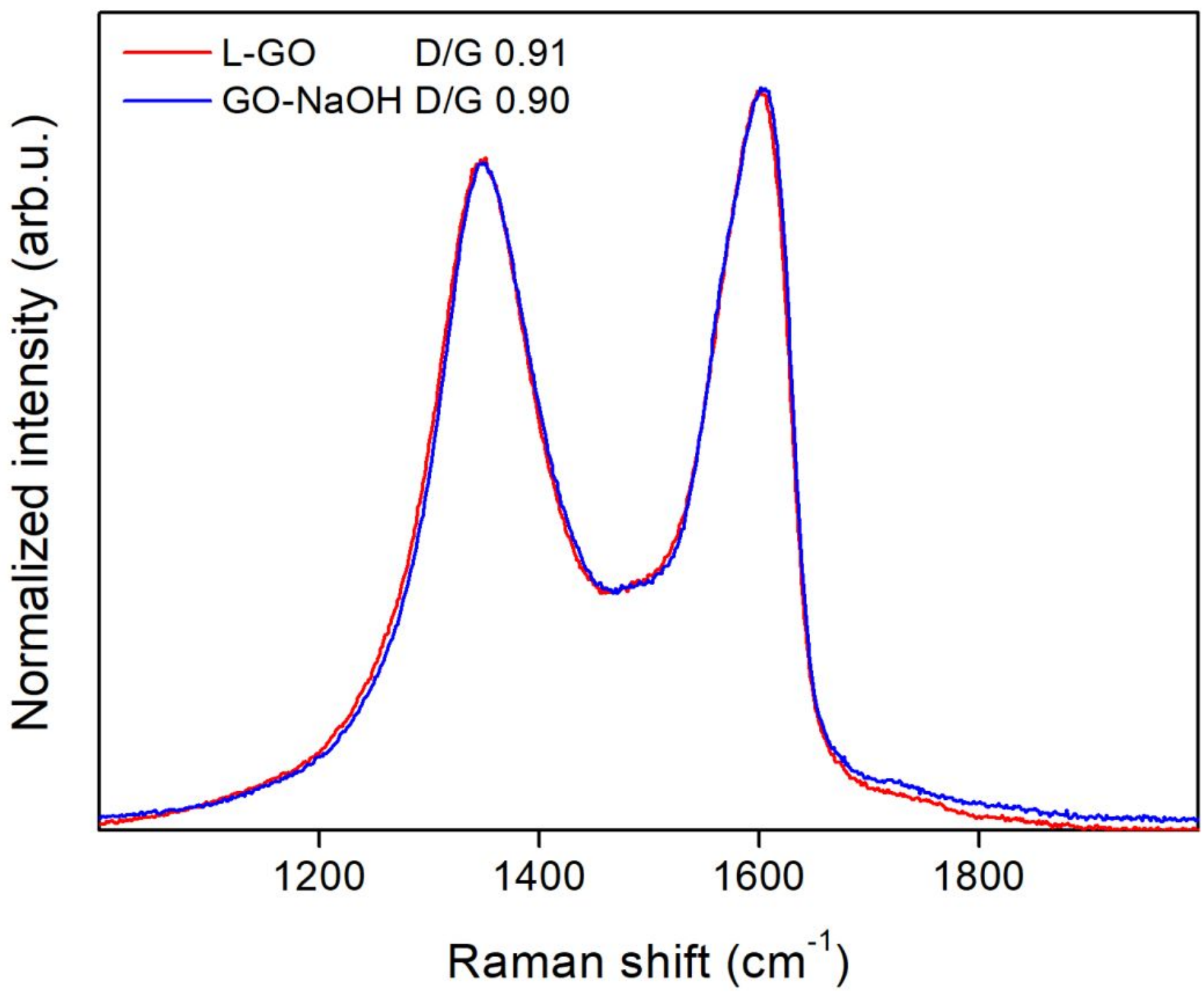

Figure S2. Raman spectra of L-GO (red line) and GO-NaOH control samples (blue line) with corresponding I_D/I_G ratio. Spectra are normalized with respect to the intensity of the G band.

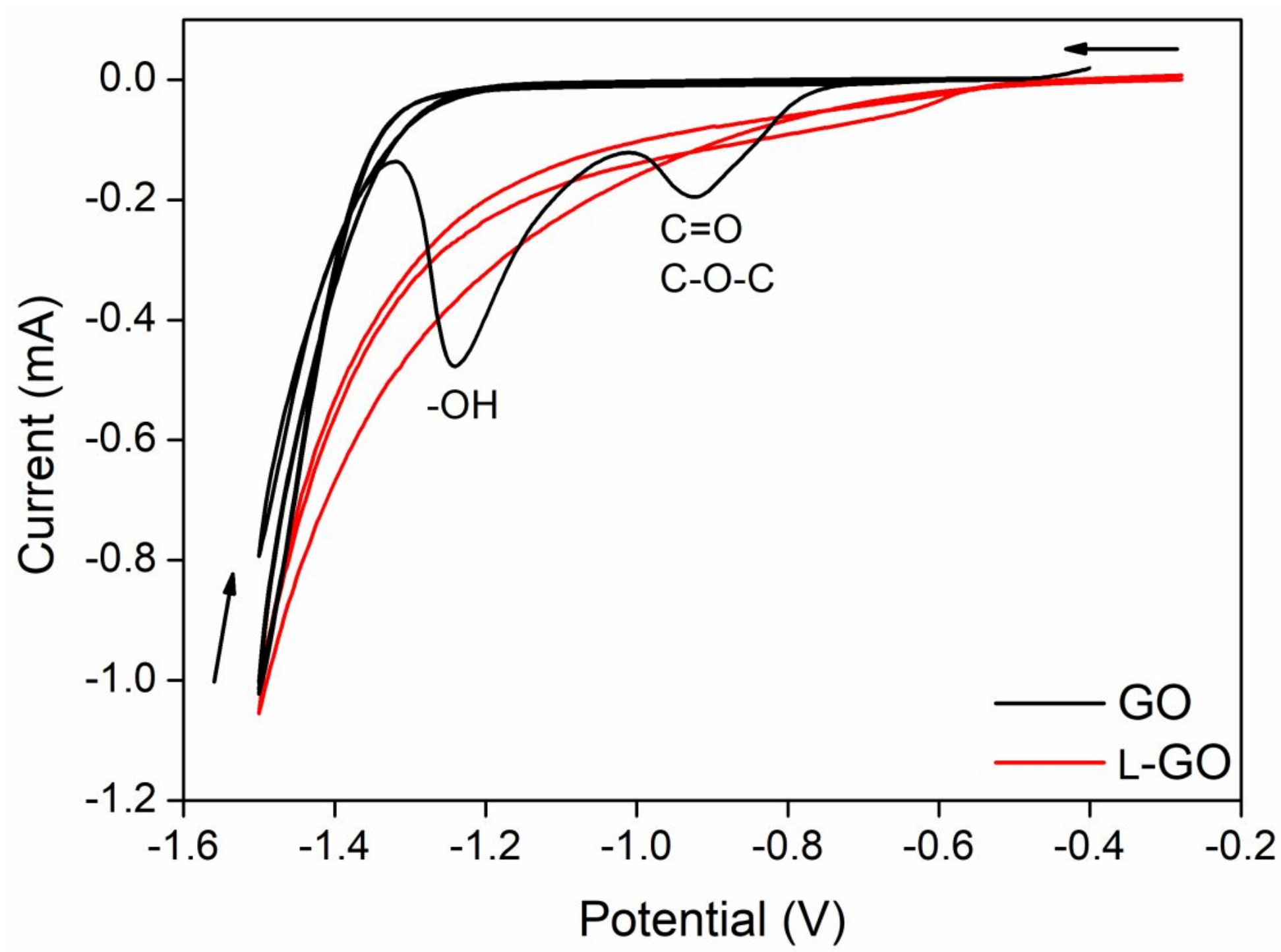

Figure S3. Cyclic voltammetry (CV) of a pristine GO (black line) and L-GO (red line) samples investigated in this work.

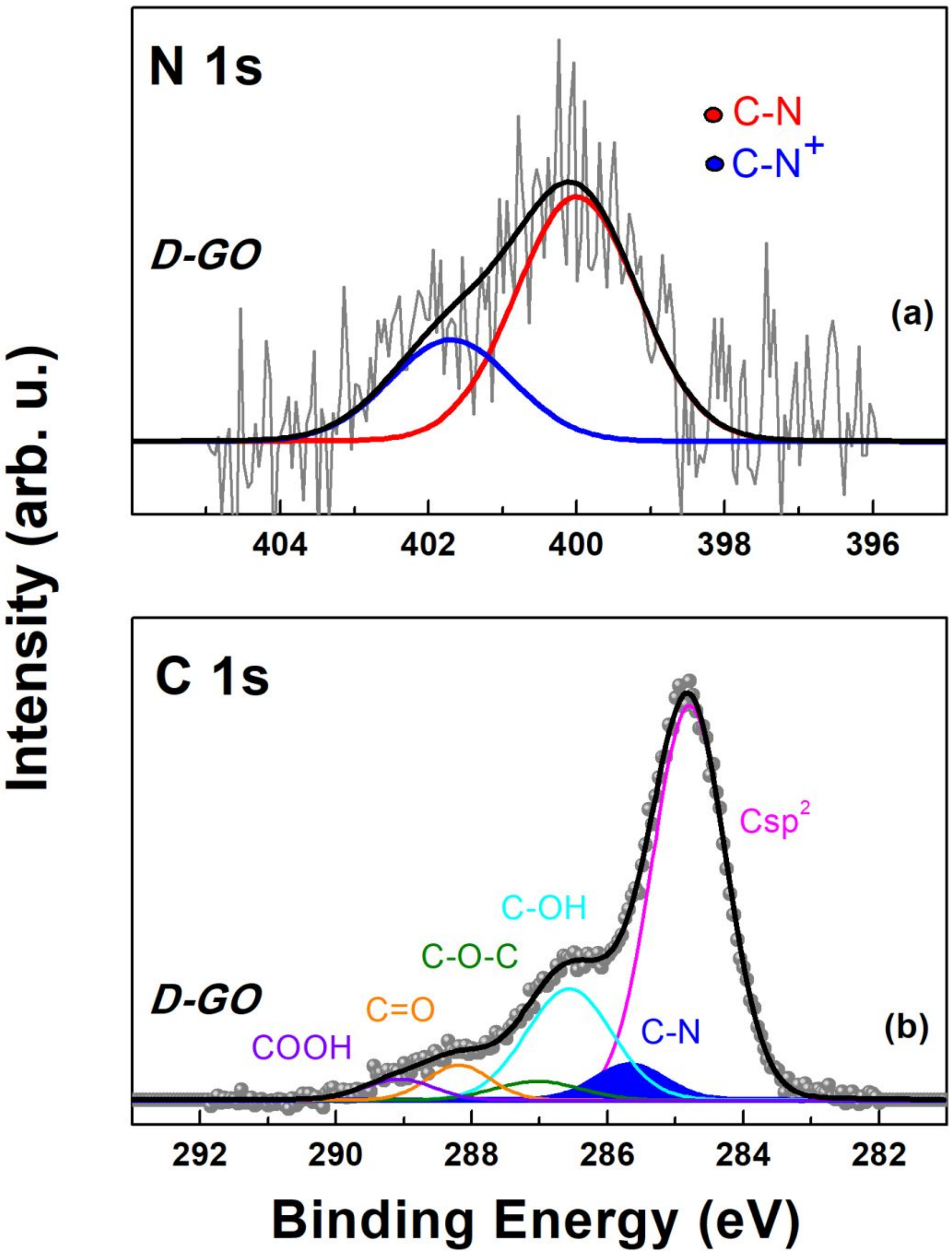

Figure S4. High-resolution (a) N 1s and (b) C 1s XPS spectra of D-GO. Results of curve-fitting are reported as coloured curves.

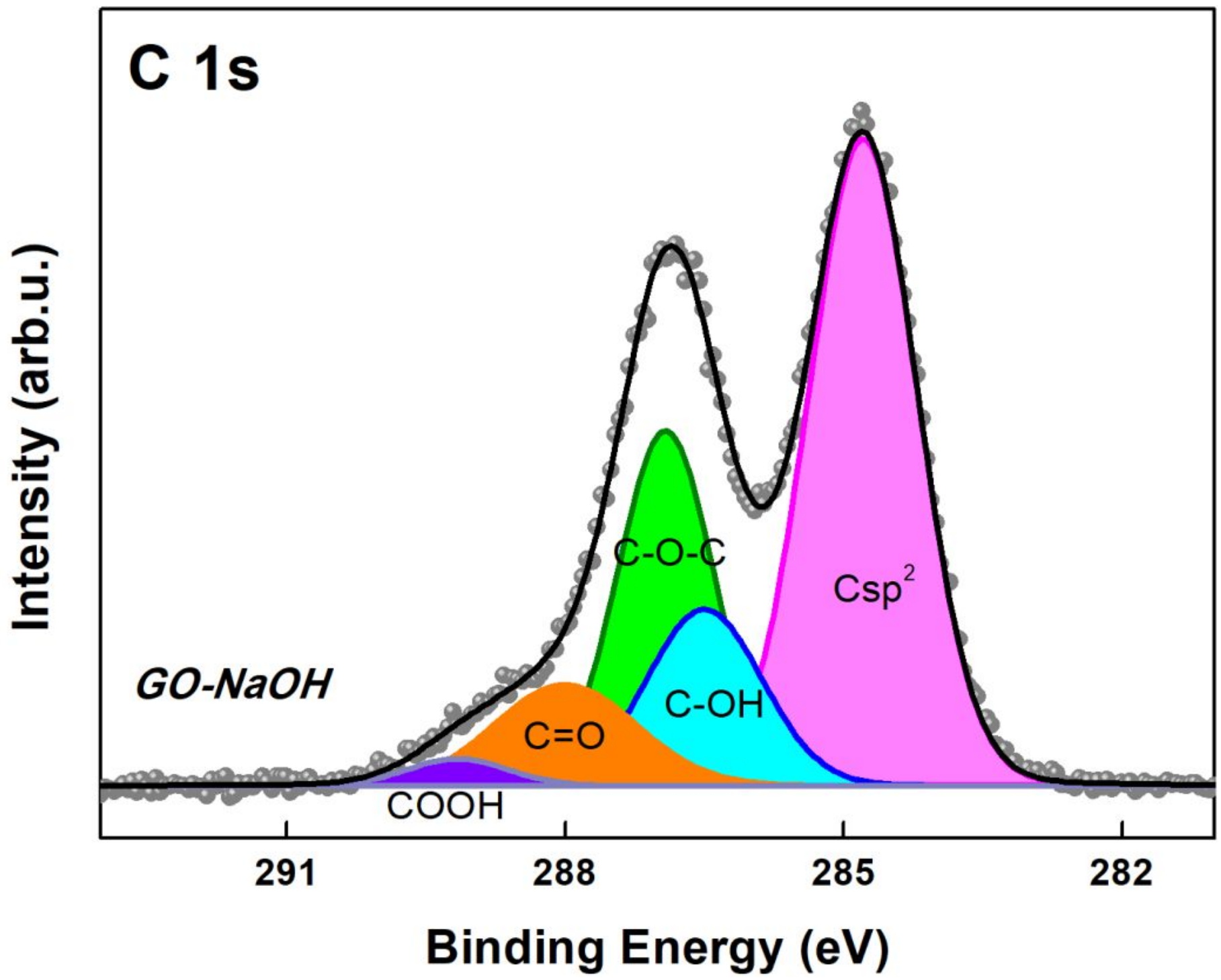

Figure S5. High-resolution C 1s spectrum of GO-NaOH. Components of curve-fitting are reported as coloured curves.

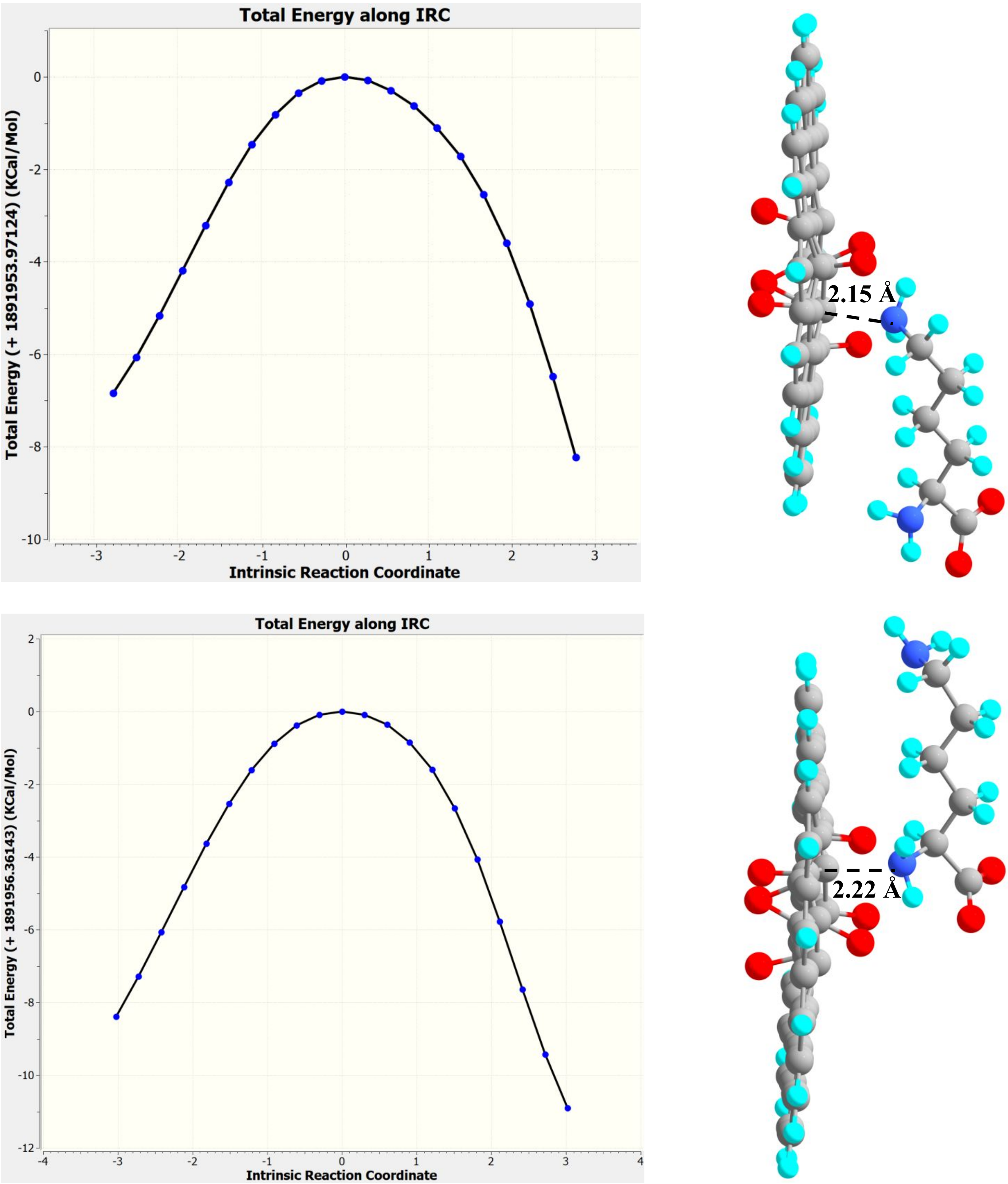

Figure S6. Intrinsic Reaction Coordinate (IRC) calculation of the TS of the epoxy ring opening by ε - (**a**) and α-amino group (**b**). 10 steps with a default step size of 0.10 bohr were done in both the backwards and forward directions. In the calculated TS, the C-N bond distance is 2.15 Å when the epoxy ring reacts with the ε-nitrogen atom, and of 2.22 Å when it reacts with the α-nitrogen atom. Both IRC enlighten the formation of C-N bond followed by the opening of the epoxy ring, thus leading to the charge-separated intermediate (INT-1). The IRC calculations confirm that, for both reaction pathways, the transition state correctly connects the reactants to intermediate INT-1.

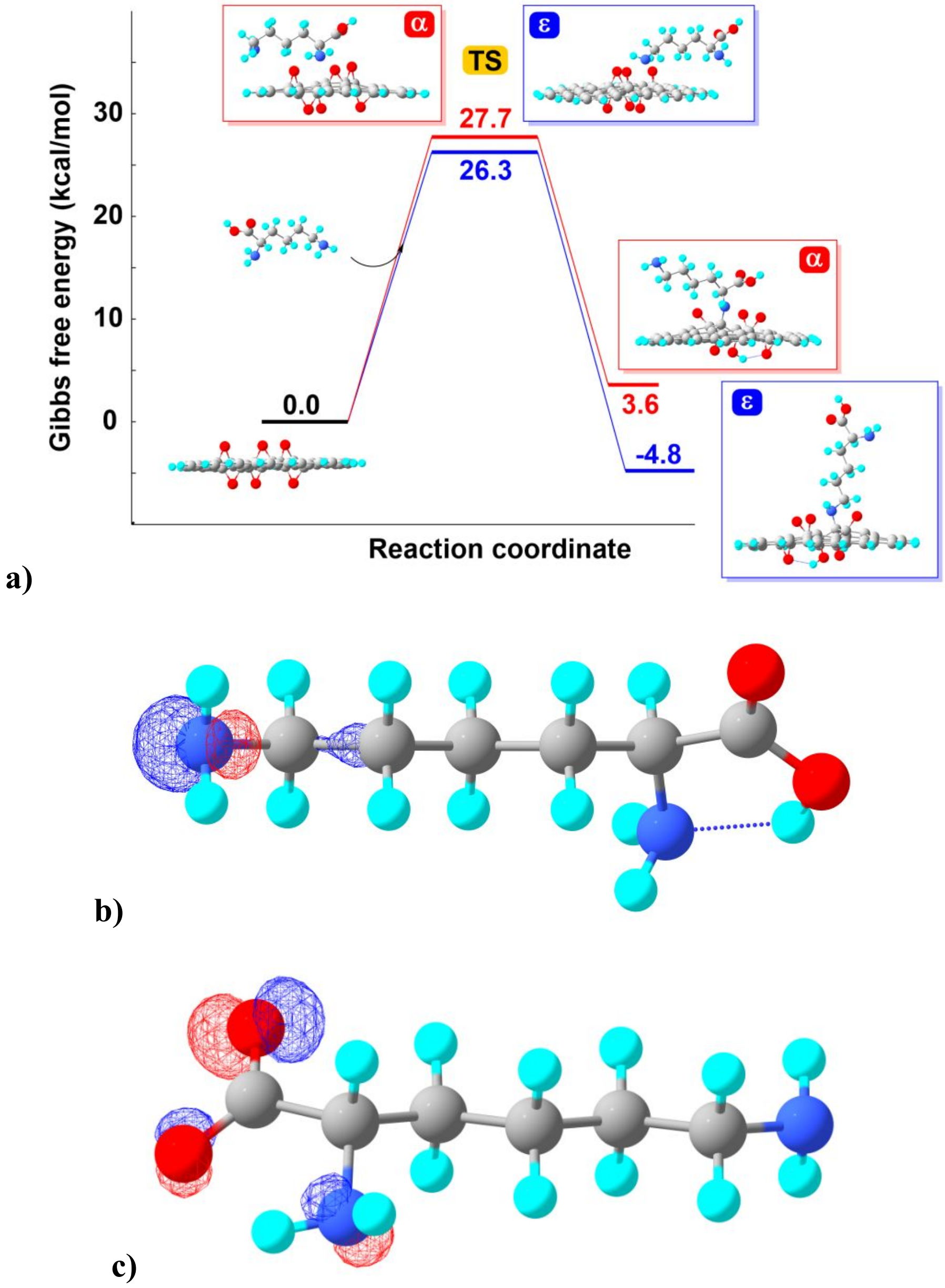

Figure S7. Lysine can react with GO through either the α-amino group or the ε-amino group. a) When the carboxyl group of lysine is considered as protonated, functionalization through the ε-amino group is thermodynamically more favorable. In contrast, when the negatively charged form (COO^-) is used, functionalization through the α-amino group becomes slightly more stable (Figure 4). It is therefore clear that steric effects alone, if operative, cannot fully account for this behavior. What changes is the nucleophilicity of the two amino groups as a function of the molecular charge state. b) In the neutral molecule, the formal charges (calculated by NBO analysis) are nearly identical on the two nitrogen atoms (−0.88). Furthermore, visualization of the HOMO reveals that it is primarily localized on the ε-nitrogen atom. Therefore, steric effects (which are likely minimal) together with electronic population effects both tend to make the ε-nitrogen more nucleophilic than the α-nitrogen. c) When negatively charged lysine is considered, the formal charge (calculated by NBO analysis) on the α-nitrogen is found to be greater (−0.91) than that on the ε-nitrogen (−0.88). In addition, analysis of the HOMO shows that the electron density is now shifted toward the α-nitrogen. Finally, there is no possibility of forming intramolecular hydrogen bonds involving the α-nitrogen. As a result, both electronic and conformational effects contribute to making the α-nitrogen slightly more nucleophilic than the ε-nitrogen in the negatively charged form of lysine.

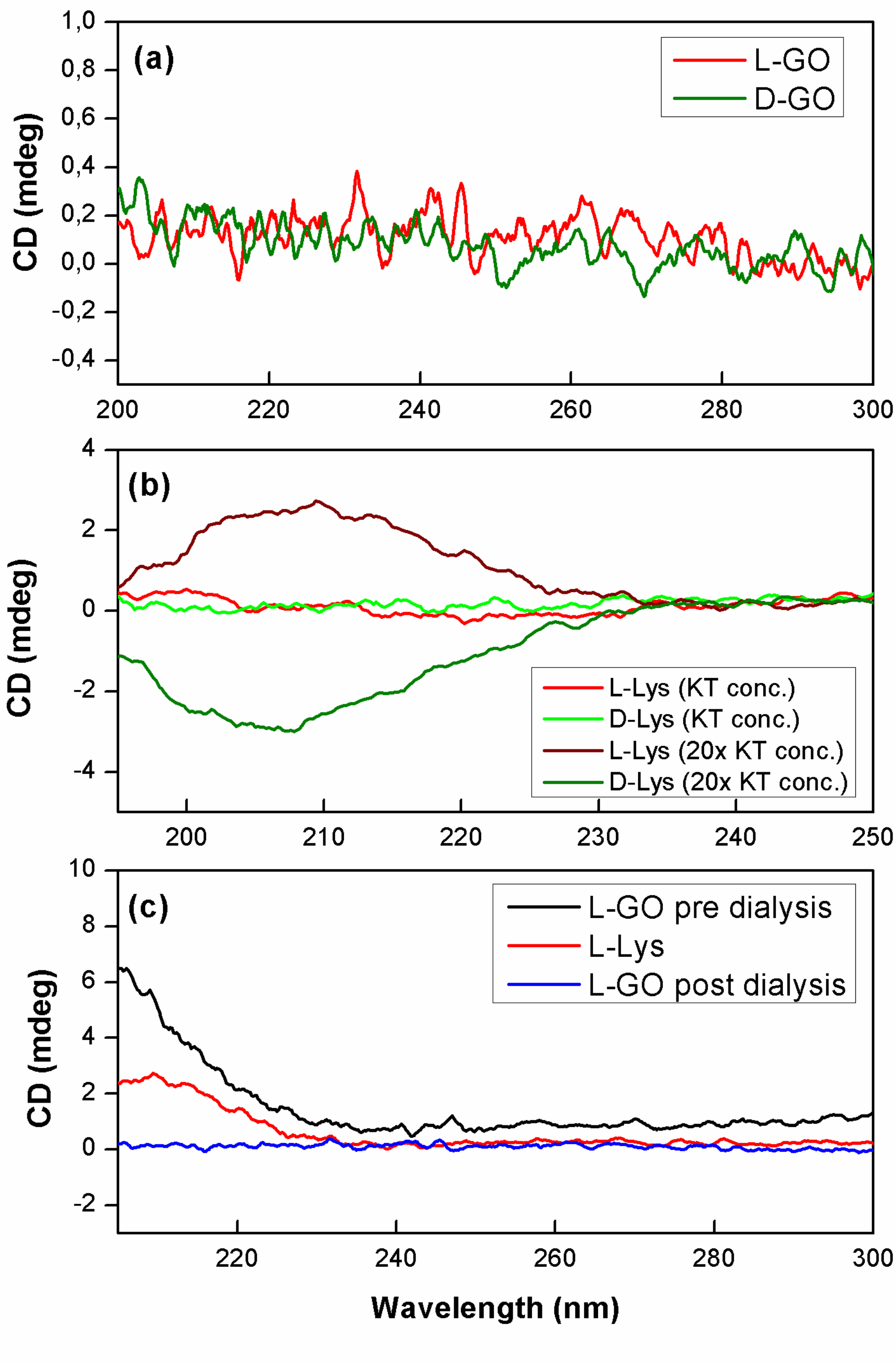

Figure S8. CD spectra of (a) L- and D-GO solution (0.06 mg mL^{-1}); (b) Pure lysine solutions, both L- and D- enantiomers, at the measured KT concentration (7.9 µM) and a 20x concentration (0.16 mM); (c) L-GO solution (0.06 mg mL^{-1}) pre- and post-dialysis. The CD spectrum of L-Lys is also reported for reference.

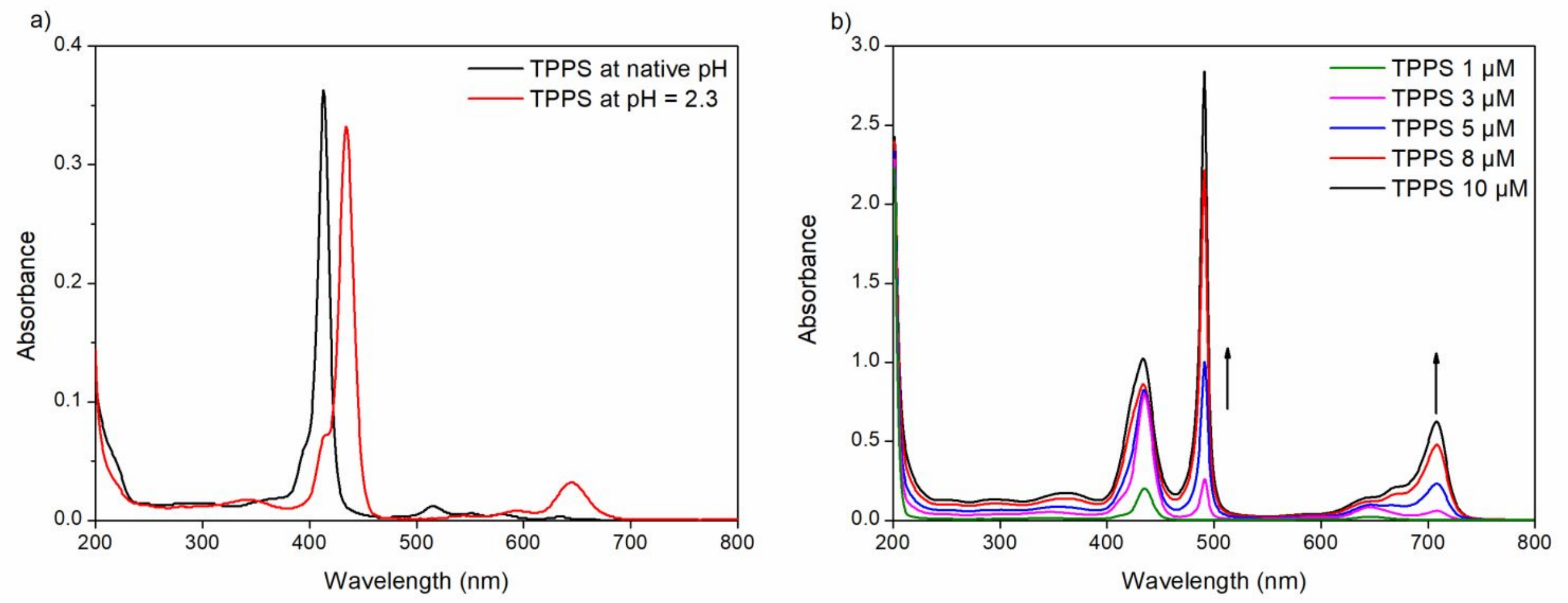

Figure S9. a) UV-Vis spectra of aqueous solutions of TPPS (1 μM) at different pH and b) UV-Vis spectra of TPPS at different concentrations recorded in acidic aqueous medium (0.1 M HCl) containing 0.3 M NaCl. Arrows indicate the appearance of the diagnostic spectroscopic signals (Soret and Q bands) of the J-aggregates as the TPPS concentration increases.

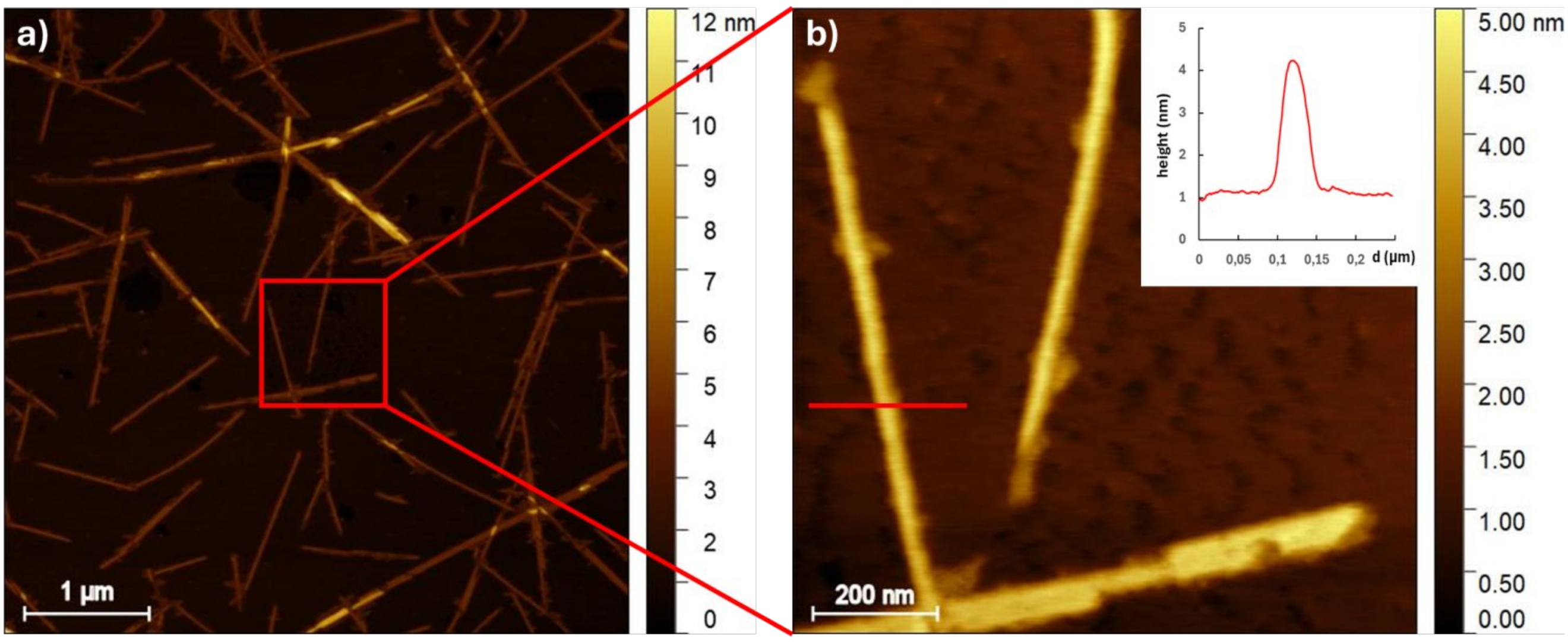

Figure S10. Tapping mode AFM images of the J-aggregates of TPPS (6 μM) formed in water and in the presence of HCl (0.1 M) and NaCl (0.3 M): (a) 5 μm × 5 μm, (b) 1 μm × 1 μm. Inset: height profile of a single fibre corresponding to the red bar in (b).

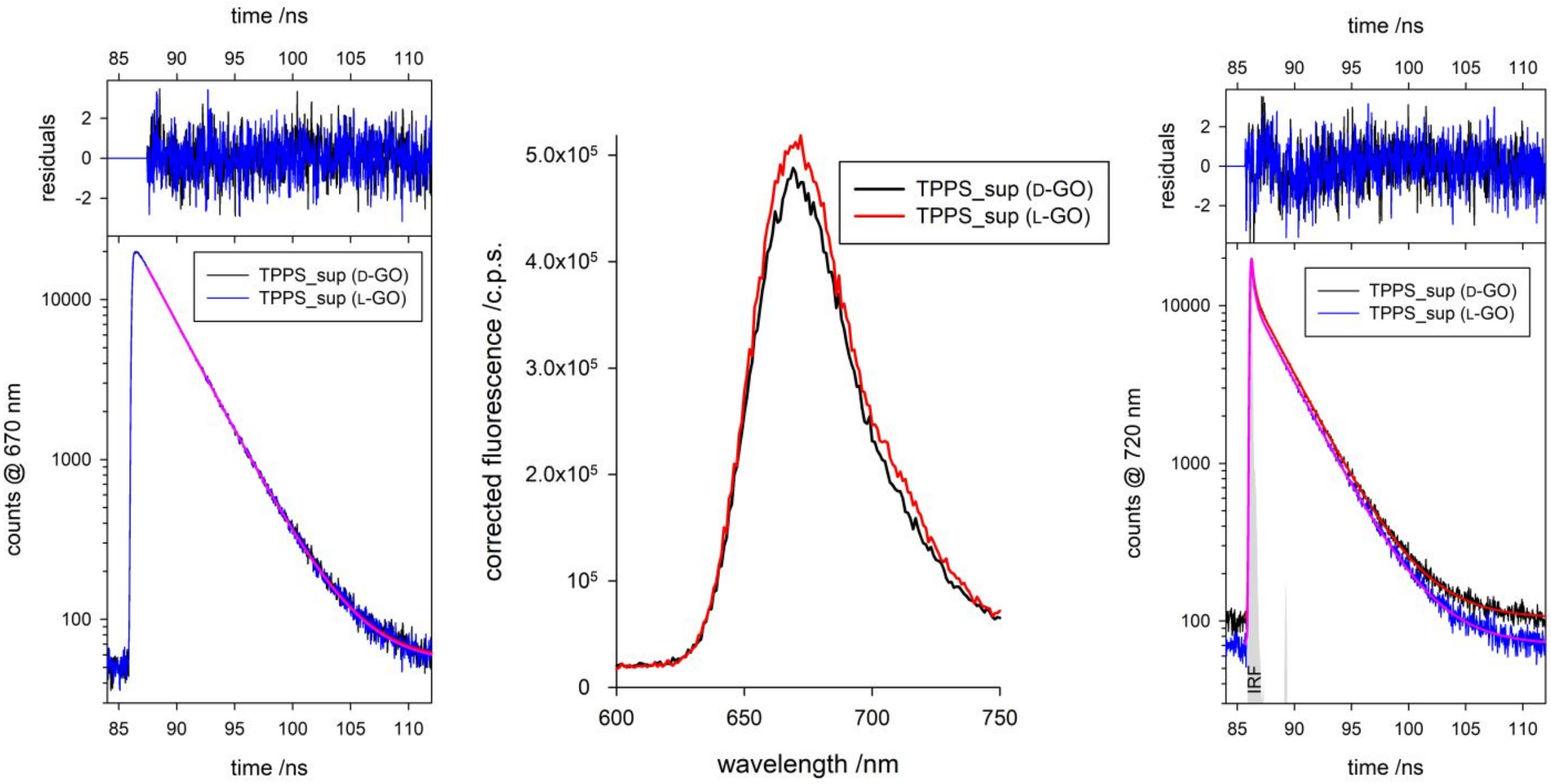

Figure S11. Center: Emission spectra of TPPS_sup (D-GO) (black trace) and TPPS_sup (L-GO) (red trace) obtained exciting the samples at 378 nm (optical path length: 0.3 × 0.3 cm; T= 25 °C; slits 2.0/2.0 nm). Left: Fluorescence lifetime measurements (TPPS_sup (D-GO), black trace; TPPS_sup (L-GO), blue trace) at 670 nm globally fitted (magenta lines; residuals in the upper plot) to a single exponential ($\tau = 3.19 \pm 0.01$ ns). Right: Fluorescence lifetime measurements (TPPS_sup (D-GO), black trace; TPPS_sup (L-GO), blue trace) at 720 nm fitted to a reconvolution function (magenta and red lines; residuals in the upper plot) of the Instrument Response Function (grey shaded area; IRF) to the sum of two exponentials ($\tau_{fast} = 0.22 \pm 0.01$ ns – 54±1 %; $\tau_{slow} = 3.17 \pm 0.02$ ns – 46±1 %).

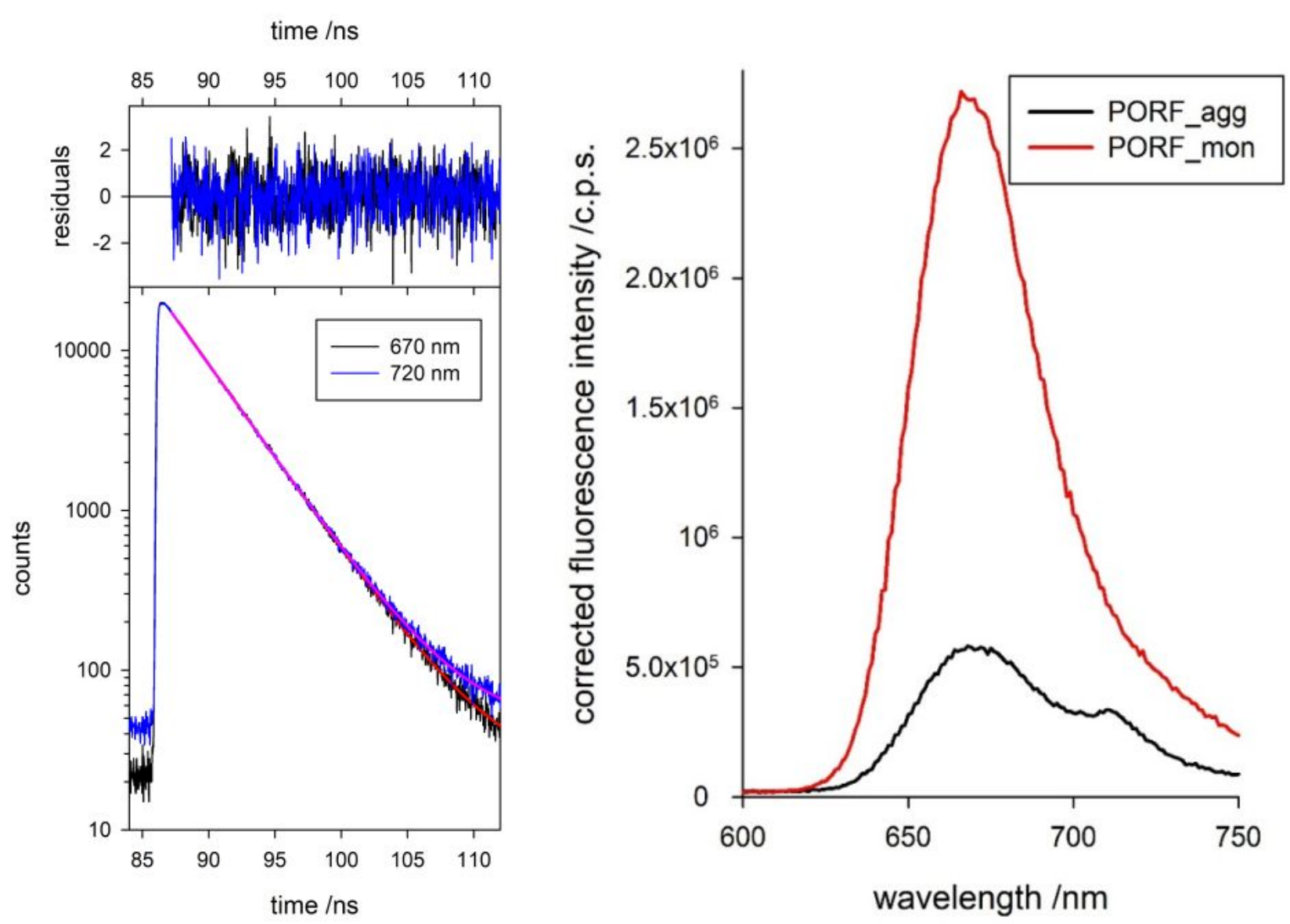

Figure S12. Center: Emission spectra of aggregated (“PORF_agg”, TPPS 6 μM, 0.1 M HCl, 0.3 M NaCl) and monomeric (“PORF_mon”, TPPS 6 μM, 0.1 M HCl) TPPS obtained exciting the samples at 378 nm (optical path length: 0.3 × 0.3 cm; T= 25 °C; slits 2.0/2.0 nm). Left: Fluorescence lifetime measurements on monomeric TPPS acquired at 670 nm (black trace) and at 720 nm (blue trace) globally fitted (residuals in the upper plot) to a single exponential (τ = 3.72±0.01 ns). Right: Fluorescence lifetime measurements on aggregated TPPS acquired at 670 nm (black trace) fitted to a single exponential (τ = 3.18±0.01 ns) and at 720 nm (blue trace) fitted (residuals in the upper plot) to a reconvolution function of the Instrument Response Function (grey shaded area; IRF) to the sum of two exponentials (τ_{fast} = 0.26±0.01 ns – 80 %; τ_{slow} = 3.12±0.02 ns – 20 %).

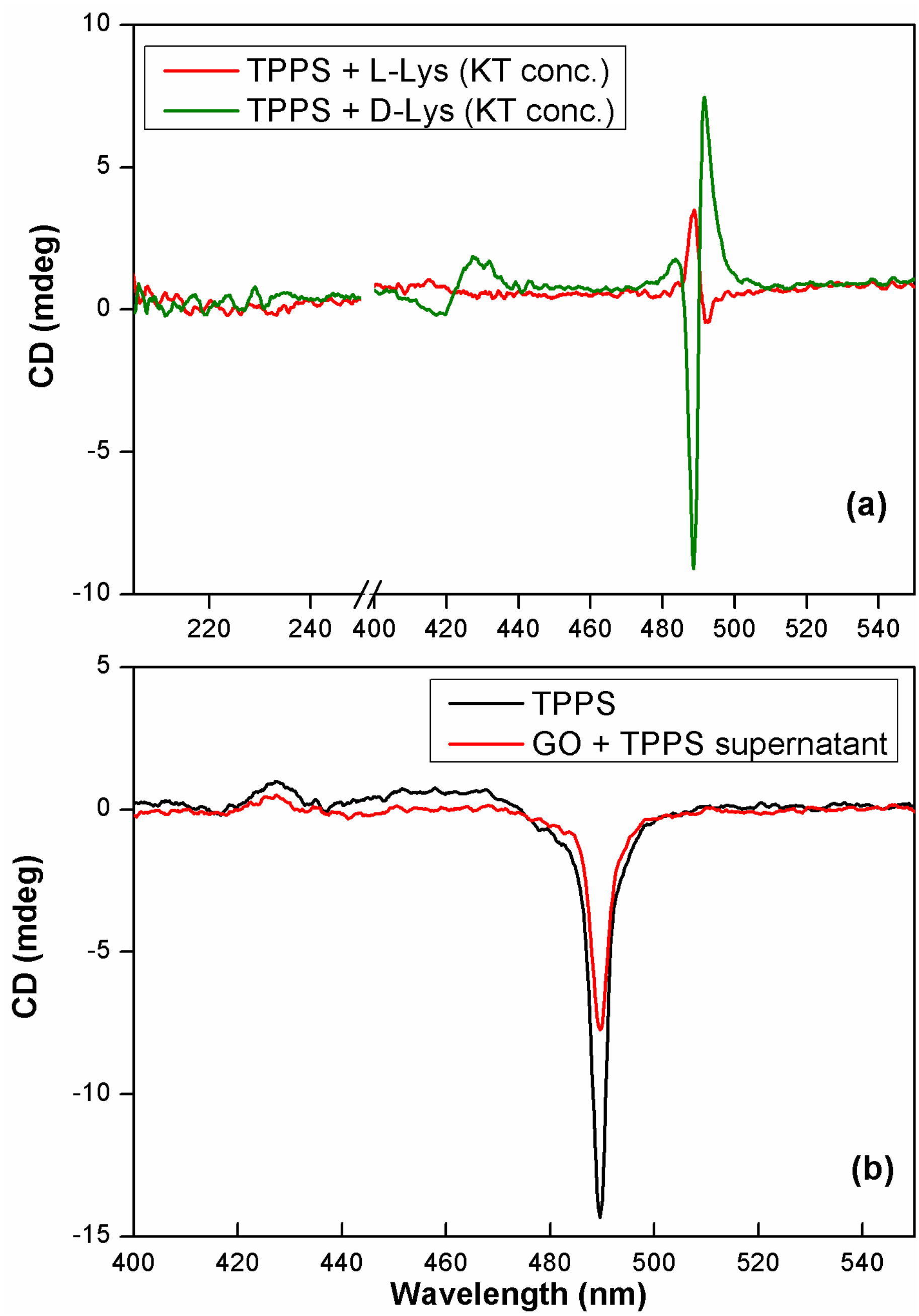

Figure S13. CD spectra of (a) Pure lysine solutions, both L- and D- enantiomers, at the measured KT concentration (7.9 μM) + TPPS in the conditions for the preparation of supernatant samples (see main text); (b) GO + TPPS vs. pure TPPS, both obtained in the same conditions for the preparation of supernatant samples (see main text).

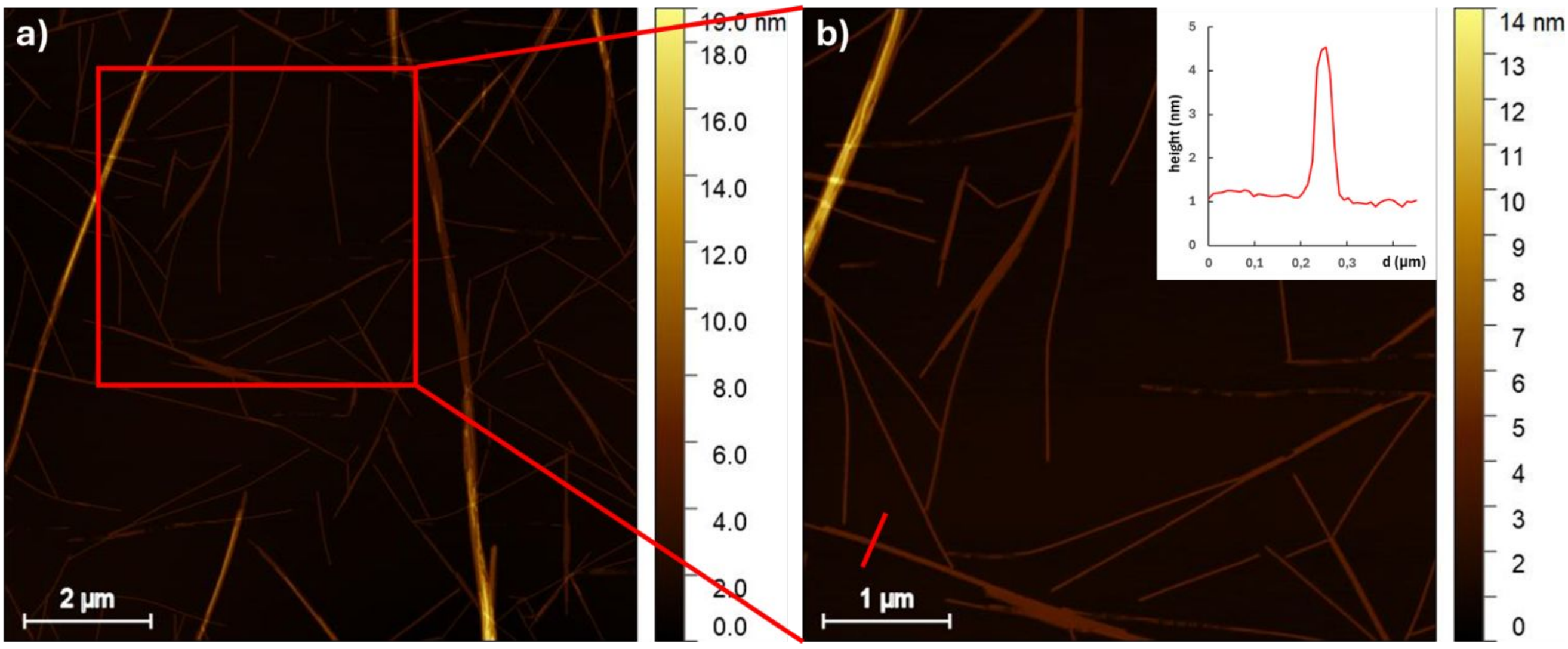

Figure S14. Tapping mode AFM images of the J-aggregates of TPPS_sup (D-GO) sample: (a) 10 µm × 10 µm, (b) 5 µm × 5 µm. Inset: height profile of a single fibre corresponding to the red bar in (b).
